# Lighting up necrotic neurons from traumatic brain injury with magnetic quantum dots to decode the cellular origin of MRI

**DOI:** 10.1126/sciadv.aej0686

**Published:** 2026-11-04

**Authors:** Mingxia Jiao, Xin Zhao, Jingyun Wang, Wanru Tian, Yao Li, Xuan Cheng, Kun Zhang, Xiliang Luo, Peisen Zhang, Mingyuan Gao

**Affiliations:** ^1^ Key Laboratory of Optic-electric Sensing and Analytical Chemistry for Life Science, MOE, College of Chemistry and Molecular Engineering, Qingdao University of Science and Technology, Zhengzhou Road 53, Qingdao 266042, China.; ^2^ School of Life Sciences, Biomedical Basic Research Center of Jiangsu, Soochow University, Suzhou 215123, Jiangsu, China.; ^3^ Suzhou XinYing biomedical Company Limited, Suzhou 215123, China.

## Abstract

The diagnosis of traumatic brain injury (TBI) remains heavily constrained by the limitations of conventional imaging techniques in visualizing the pathological events that determine the disease progression and outcome. To address this clinical challenge, we report a manganese-doped near-infrared quantum dot (QD) to intrinsically integrate the highly sensitive optical imaging with clinical compatible magnetic resonance imaging (MRI). Taking the advantages of optical imaging powered by whole-brain clearing and cellular resolution three-dimensional (3D) panoptic imaging, in combination with the systematic in vitro cell studies on different types of cells involved in TBI, we disclosed the interpretable pathology reflected by TBI imaging through the highly sensitive fluorescence of magnetic QDs, i.e., after systemic administration, the nanoprobes were extravasated through the compromised blood-brain barrier and then selectively phagocytosed by activated microglia, which subsequently migrated into the regions rich in dead neurons and neuronal debris. This microglia-mediated tropism decodes the signal origin of MRI enhanced by the nanometer-sized contrast agent, thereby transforming the MRI from an anatomical tool into a pathological reporter. A biocompatible Fe_3_O_4_ nanoparticle of similar size was also compared with the magnetic QD mentioned above for validating the translational merits of nanoparticles in MRI diagnosis of TBI.

## INTRODUCTION

Traumatic brain injury (TBI) represents a major global health challenge, contributing to substantial mortality and long-term disability worldwide. It encompasses complex pathological conditions resulting from external mechanical forces to the head, leading to immediate and progressive damage to brain tissue (*1*–*5*).

The clinical management of TBI is profoundly complicated by difficulties in accurate and timely diagnosis (*6*–*8*). Conventional imaging techniques, particularly computed tomography (CT), are helpful for identifying life-threatening hematomas and skull fractures but notoriously incapable of showing diffuse axonal injury or microstructural soft tissue pathologies that are hallmarks of many TBI cases (*9*, *10*). Although magnetic resonance imaging (MRI) is more sensitive than CT in soft tissue imaging, its sensitivity remains limited in reflecting the early and subtle pathological alterations caused by TBI (*11*–*13*). This inability impedes timely intervention of TBI, such as the administration of neuroprotective agents within a critical therapeutic window. It also hinders accurate prognosis, leading to suboptimal rehabilitation (*14*, *15*). Furthermore, the lack of precise information on neuroinflammation and neurodegeneration frustrates the development of efficacious therapeutic strategies (*16*), resulting in mismanagement of the patients, inefficient allocation of healthcare resources, and ultimately unsatisfied neurological outcomes, as well as increased long-term disability rates.

The pathological aftermath of TBI involves a complicated cascade of cellular and molecular events involving diverse cell populations (*17*–*19*). The initial mechanical insult triggers primary damage, which is rapidly followed by a detrimental secondary injury phase that is characterized by excitotoxicity, pervasive neuroinflammation, oxidative stress, and widespread cell death (*20*–*22*). Neurons undergo apoptosis and necrosis, the blood-brain barrier (BBB) is compromised due to dysfunction and death of vascular endothelial cells, microglia, etc., and the resident immune cells of the brain become rapidly activated. The activated microglia then migrate to the lesion site to clear cellular debris and dead cells and meanwhile release cytotoxic cytokines and propagate damaging inflammatory responses (*23*, *24*). Crucially, the spatial distribution and interplay of these pathological processes involving different cell types determine the progression and final outcomes of the injury. Therefore, spatiotemporally visualizing these specific cellular and molecular events is paramount for understanding the pathogenesis and precise diagnosis of TBI as well. However, the current clinical imaging modalities remain largely confined to the macroscopic, anatomical level, failing to provide essential cell-type-specific information.

Nanoparticles have emerged as a promising platform for addressing these diagnostic limitations (*25*–*29*). The inherent physical properties of functional nanomaterials, particularly magnetic quantum dots (QDs), allow for a perfect combination of optical imaging with MRI, which leverages the synergistic strengths of each imaging technique to bridge fundamental research and clinical application (*30*–*32*). Because optical imaging is much more sensitive than MRI, and it is thus very suitable for reflecting TBI through perfusion and even specific interactions of QDs with various types of TBI-associated cells, which will, in turn, help understand the pathologic origin of the enhanced MRI that is clinically compatible.

Toward pathology-interpretable diagnosis of TBI, a biocompatible nanoprobe based on Cd-free QDs was designed herein. By doping the near-infrared (NIR)–emitting QDs with Mn^2+^ ions, we developed an optical/MR dual-modality imaging nanoprobe for noninvasively mapping the TBI-associated pathological cells to finally establish a cellular-level diagnostic benchmark for TBI (Fig. 1). The ultimate goal is to use the mechanistic insights gained from the optical imaging to interpret and validate the MRI signals enhanced by nano contrast agents that are emerging as clinically translatable MR contrast agent. In short, the Mn-doped QDs were synthesized and characterized. Their interactions with key central nervous system cells were investigated in vitro, followed by in vivo animal experiments in a mouse model of TBI to show the spatial distribution TBI-associated cells for better understanding the MRI of TBI enhanced by nanoprobes, e.g., Fe_3_O_4_ nanoparticles of similar size, to show what one learns from the magnetic QDs is extendable to other types of nanoprobes.

**Fig. 1.**
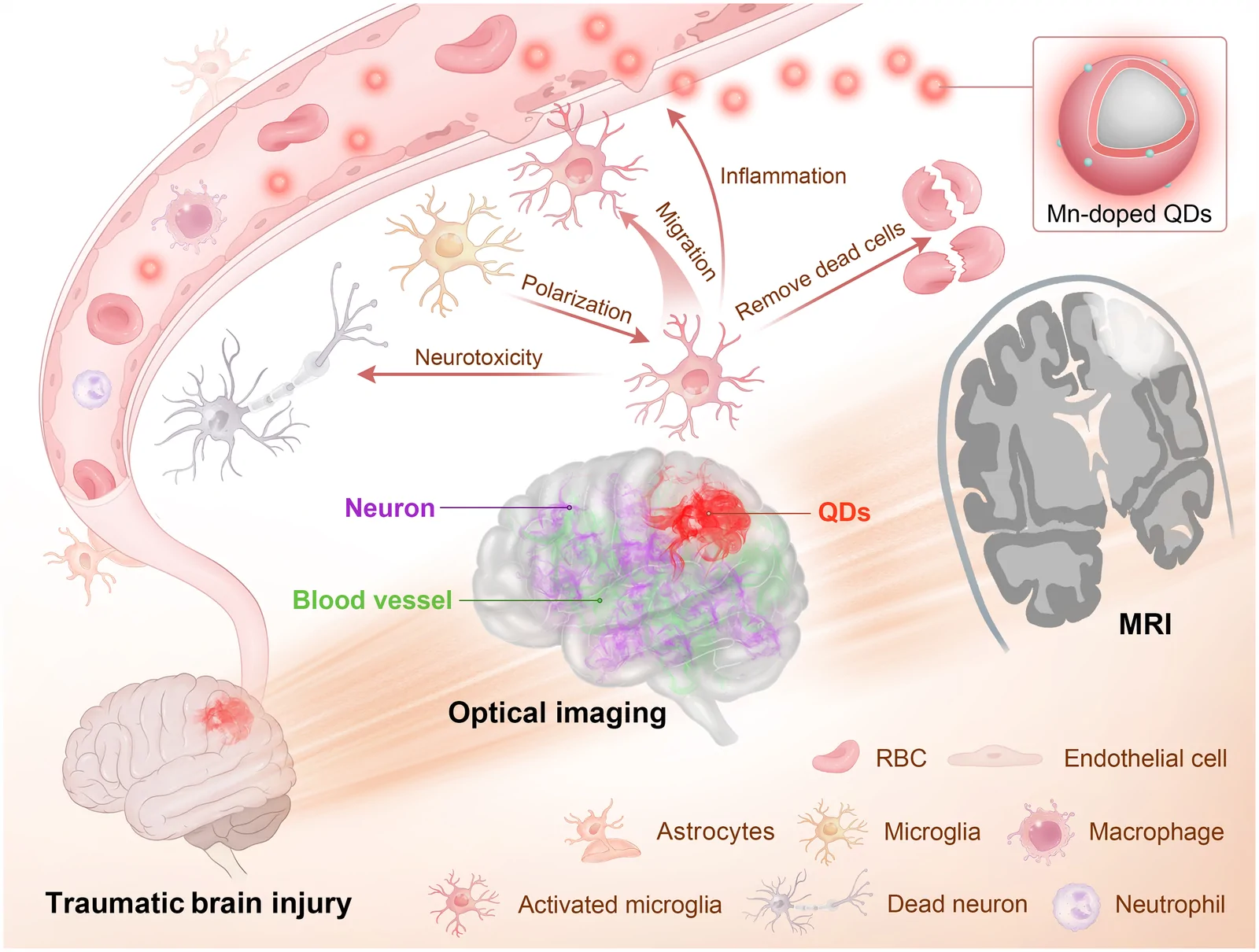
Schematic illustration of a biocompatible nanoprobe for integrated optical and MRI based on Mn-doped NIR QDs. Highly sensitive optical imaging elucidates the in vivo interactions of the nanomaterials, which revealed the cellular mechanism of the *T*_1_-weighted MRI contrast enhancement, thereby transforming MRI signals from nonspecific anatomical indicators into reporters of underlying cellular pathology. This approach establishes a generalizable framework for cellular-level interpretation of MRI, advancing TBI imaging from anatomical assessment toward a specific, pathology-informed modality with direct clinical translation potential. The figure was produced by the GIMP software.

## RESULTS AND DISCUSSION

### Mn-doped core@shell QDs

Zinc copper indium sulfide (ZCIS) QDs emitting NIR light were synthesized directly in aqueous phase, and a ZnS shell was epitaxially grown on the ZCIS core to improve the photoluminescence quantum yield (PLQY) and stability of the QDs. Although the medical application of optical imaging is limited by the tissue penetration depth of optical signals, the extremely high sensitivity makes it suitable for timely visualizing the biomedical events of interest, particularly with the aid of NIR-emitting QDs containing no toxic heavy metal ions. However, with respect to clinical translation, MRI is apparently more compatible and translatable. Therefore, paramagnetic manganese ions were incorporated to endow ZCIS@ZnS QDs with MRI capability to finally obtain a NIR fluorescence/MR dual-modality imaging agent. To minimize the quenching impact of the magnetic ion, Mn^2+^ ions were selectively doped in the ZnS shell rather than QD core to spatially separate the magnetic ions from the luminescent center. The magnetic QDs finally obtained were denoted as ZCIS@ZnS:Mn. The doping level was defined as the molar percentage of Mn among total metal elements and tuned by the feed ratio of Mn precursor. The actual Mn doping molar levels were determined by inductively coupled plasma optical emission spectrometry (ICP-OES) (table S1). As shown in Fig. 2A, the exciton absorption and fluorescence are gradually red-shifted against the Mn doping level, accompanied by a gradual decrease in the PLQY, i.e., from 11.3% for the undoped QDs to 9.0% for QDs with a doping level of 4.6%, indicating successful doping of Mn^2+^ ions into the ZnS shell. This can find more supportive evidences from PL decay dynamics and lifetime studies (fig. S1 and tables S2 and S3). Although the PL intensity of QDs was slightly decreased upon Mn doping, the fluorescence intensity was still among the top of those for aqueous Cd-free QDs with emission at similar wavelength (fig. S2). Moreover, in comparison to direct Mn doping into the QD core, doping magnetic ions into the shell resulted in a much lesser loss of fluorescence efficiency (fig. S3). In addition, the broad excitation range (fig. S4) and the excitation-independent emission (fig. S5) make magnetic QDs with shell doped particularly suitable for NIR fluorescence imaging.

**Fig. 2.**
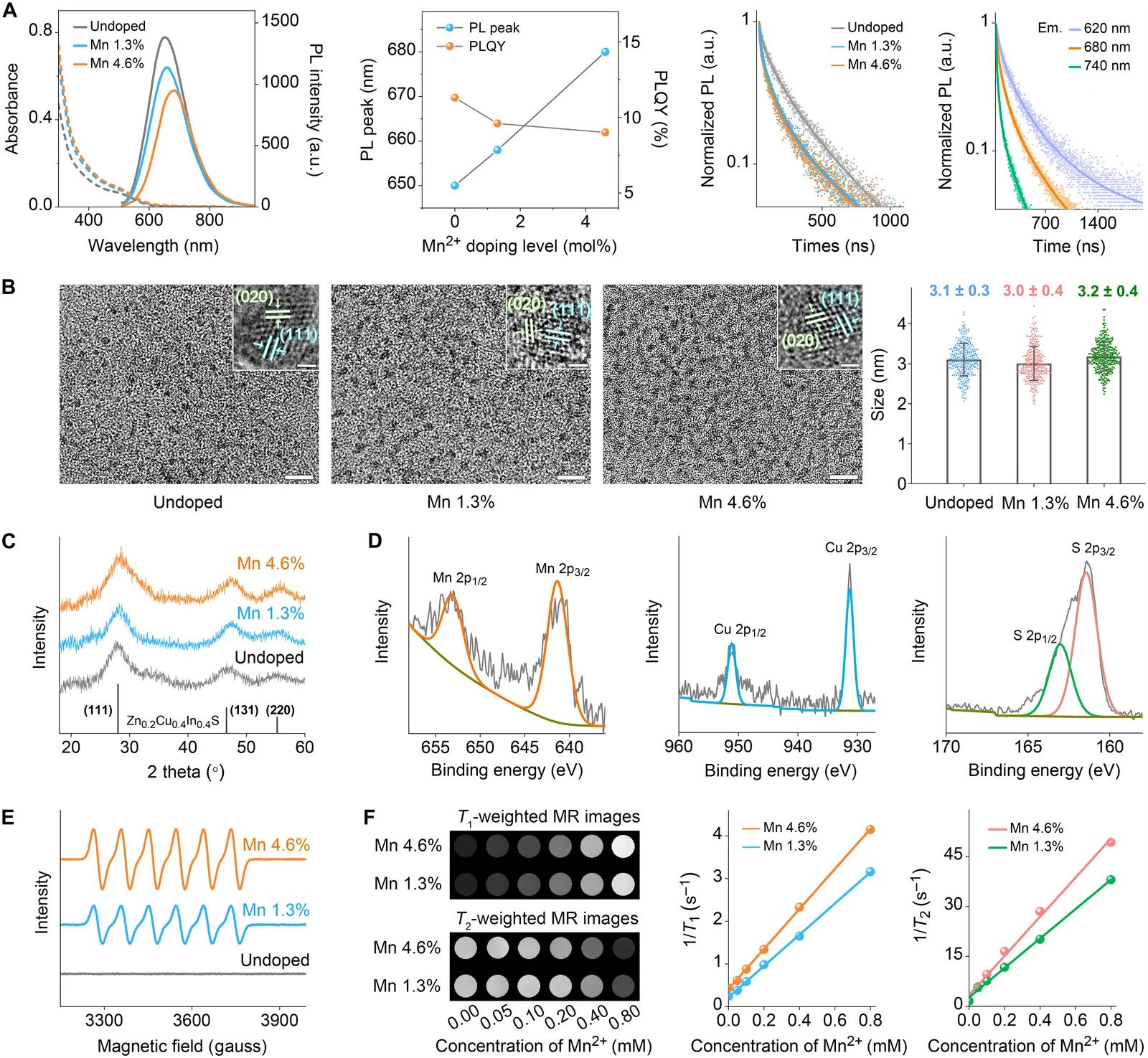
Optical, structural, and magnetic properties of Mn-doped ZCIS@ZnS QDs. (**A**) PL emission, absorption, and evolutions of the PL peak and PLQYs, and time-resolved PL decays of QDs with different Mn-doping levels. a.u., arbitrary units. (**B**) TEM images (scale bars, 10 nm) and size histograms of different QDs with insets shown HRTEM images (scale bars, 1 nm). (**C**) XRD patterns of different QDs with line patterns for cubic Zn_0.2_Cu_0.4_In_0.4_S (JCPDS card no. 47-1370). (**D**) High-resolution XPS spectra of Mn, Cu, and S elements with fitting results. (**E**) EPR patterns of different QDs. (**F**) *T*_1_- and *T*_2_-weighted MR images of aqueous solutions containing different QDs of gradient concentrations and plots of 1/*T*_1_ and 1/*T*_2_ versus the concentration of Mn overlaid with linear fitting curves for extracting the longitudinal and transverse relaxivities.

Apart from the optical properties, the impacts of Mn doping on the structure and magnetic properties of QDs were also investigated. As illustrated in Fig. 2B, magnetic doping did not alter the near-spherical morphology or monodispersity of the QDs of ∼3.0 nm. However, the x-ray diffraction (XRD) patterns in Fig. 2C, being consistent with the cubic phase of Zn_0.2_Cu_0.4_In_0.4_S (JCPDS card no. 47-1370) and well in alignment with high-resolution transmission electron microscopy (HRTEM) results shown in the insets of Fig. 2B, exhibit a slight shift toward higher angles after Mn doping, indicating a successful incorporation of Mn into the lattice of ZnS (*33*). The x-ray photoelectron spectroscopy (XPS) data in fig. S6 demonstrated that the Mn content could be varied by Mn feeding ratio. Further XPS analysis identified that the valence state of Mn was of Mn(II), whereas that of Cu was of Cu(I) (Fig. 2D). With respect to sulfur, the signal mainly came from sulfide ions within the QD lattice and l-glutathione (GSH) ligands on the particle surface. Figure 2E presents the electron paramagnetic resonance (EPR) spectra of the undoped and Mn-doped QDs. After Mn doping, a six-line hyperfine splitting pattern emerges, confirming the successful incorporation of Mn^2+^ into the lattice of ZnS shell. The EPR results also indicate that the doped Mn^2+^ ions are preferentially localized near the QD surface rather than in the bulk (*34*, *35*).

Given that the doped paramagnetic Mn^2+^ ions can effectively modulate the relaxation of water protons, the magnetic QDs were expected to serve as MRI contrast agents. The detailed results in Fig. 2F suggest that all doped QDs exhibit *T*_1_ and *T*_2_ MR contrast enhancement effects, with intensity being increased against the Mn doping level. Linear fitting yielded molar longitudinal relaxivities (*r*_1_) of 3.7 and 4.7 mM^−1^ s^−1^, and molar transverse relaxivities (*r*_2_) of 44 and 59 mM^−1^ s^−1^, for the 1.3 and 4.6% Mn-doped QDs, respectively, suggesting that higher Mn^2+^ doping level is favorable for higher relaxivity.

### Biocompatibility of magnetic QDs

Biocompatibility is an essential prerequisite for in vivo applications. To improve the biocompatibility of ZCIS@ZnS:Mn QDs, the original GSH ligands were replaced with polyethylene glycol (PEG) ligands. The Fourier transform infrared (FTIR) spectrum of QDs obtained after ligand exchange (fig. S7) exhibited pronounced C─O─C asymmetric stretching vibration and C─H stretching vibration, at 1098 and 2873 cm^−1^, respectively. These variational band coincided well with those of PEG molecule, indicating a successful PEGylation. The cytotoxicity of the resulting PEGylated QDs (PEG-QDs) was systematically evaluated using Cell Counting Kit-8 (CCK-8) assays across multiple cell lines representing different cell types, e.g., fibroblasts (NIH/3T3), brain microvascular endothelial cells (bEnd-3), microglia (BV-2), and neurons (HT-22). As shown in Fig. 3A, PEG-QDs exhibit excellent biocompatibility, with cell viability above 90% for all tested cell types at a concentration as high as 30 μM, confirming that PEG-QDs have neglectable cytotoxicity. Apart from cytotoxicity, hemocompatibility is also a critical prerequisite for intravenously administered agents. Therefore, the hemolytic risk of PEG-QDs was also evaluated before further animal experiments. As shown in Fig. 3B, the hemolysis rate remains consistently low (between 0.9 and 4.0%) across a wide concentration range of 0.5 to 30 μM, which is well below the safety threshold (5%) proposed by the American Society for Testing and Materials (ASTM) (*36*). Notably, the peak blood concentration of PEG-QDs postinjection, i.e., 12.3 μM, was substantially lower than the maximum concentration used in hemolysis experiment, further supporting their low toxicity risk.

**Fig. 3.**
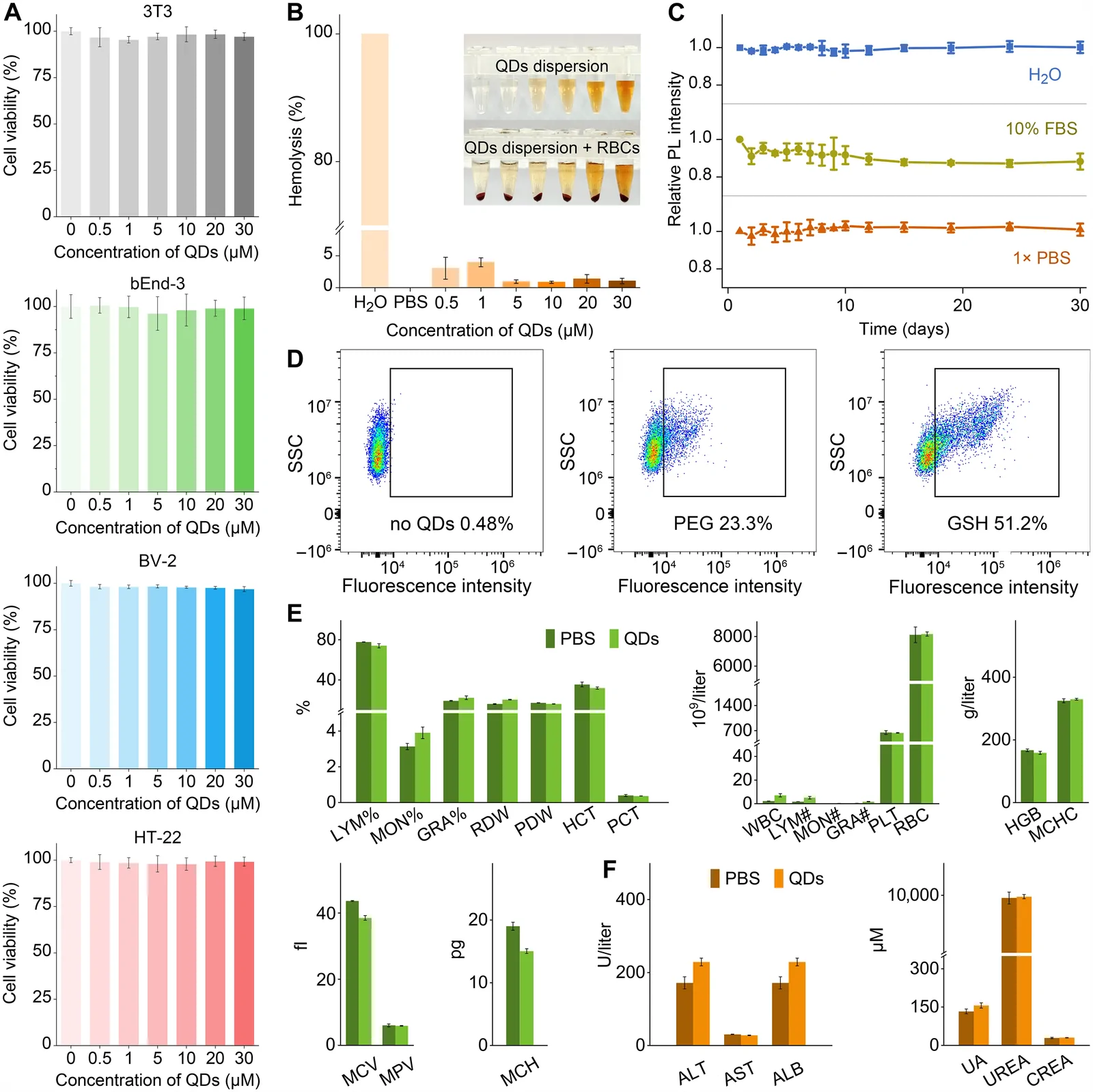
Biocompatibility evaluation of Mn-doped QDs. (**A**) Viabilities of different types of cells treated with PEG-QDs with different concentrations. (**B**) Hemolysis rate of PEG-QDs with different concentrations. Inset: The photographs of PEG-QD dispersions at different concentrations with or without RBCs after centrifugation. (**C**) Changes in the relative PL intensity of PEG-QDs in different media over a period of 30 days. (**D**) Quantitative assessment of cellular QD uptake by flow cytometry. (**E**) Complete blood count and (**F**) blood chemical test of QD-treated or PBS-treated mice (*n* = 4).

Apart from cytotoxicity and hemolysis, the stability of QD fluorescence under physiological conditions is also important for in vivo applications. Thus, the PL intensity of PEG-QDs in water, 1× phosphate-buffered saline (PBS), or fetal bovine serum (FBS) was monitored over 30 days. As depicted in Fig. 3C, the PL exhibits no obvious attenuation in intensity in all tested media, confirming an exceptional stability of the NIR fluorescence.

In the previous studies, the PEG modification was often adopted to endow nanomaterials with “stealth” characteristics, allowing them to evade recognition by the host immune system (*37*, *38*). In this study, mouse leukemia cells of monocyte macrophage RAW 264.7 was employed to evaluate such antiphagocytosis capability of PEG-QDs. As shown in fig. S8, the mouse macrophages exhibit significantly higher fluorescence after incubation with GSH-QDs than those incubated with PEG-QDs, suggesting that the cell uptake of PEG-QDs by macrophages is effectively suppressed by the surface PEGylation. This effect was quantified in Fig. 3D. In comparison to the untreated cells, ∼51.2% of macrophages show an evident fluorescence signals following the incubation with GSH-QDs, whereas this percentage drops to 23.3% for macrophages incubated with PEG-QDs, highlighting the outstanding biocompatibility of PEG-QDs, perfectly meeting the stringent requirements for in vivo imaging applications.

As an imaging agent for TBI studies, QDs should not exacerbate inflammatory responses. In this regard, intracellular reactive oxygen species (ROS) generation possibly induced by QDs was evaluated. As shown in fig. S9, no obvious difference in ROS levels was detected in QD-treated cells compared with untreated controls, indicating that QD exposure did not aggravate inflammatory responses.

Apart from the biocompatibility assessments in vitro, the effects of PEG-QDs were systemically evaluated in vivo. Specifically, the hematological and toxicological effects of PEG-QDs were examined after they were intravenously injected into in BALB/c mice by a dose of 27 mg QDs per kg body weight, with an equivalent volume of 1× PBS as a control.

As shown in fig. S10, the average body weight of the mice from the experimental group slightly decreased during the first 3 days postinjection of the QDs. It got recovered on day 6 and returned to normal level approximately after day 12. Thereafter, there was no substantial difference in body weight between the experimental and control groups. The following blood tests showed no significant differences in routine blood indices in comparison to those of healthy mice 20 days postinjection, and no signs of hemolysis or inflammation were observed, as evidenced by normal RBC, PLT, MCV, MPV, PCT, WBC, LYM, MON, and GRA levels (Fig. 3E). Furthermore, liver and kidney function remained unaffected by the QDs, with liver (ALT, AST, and ALB) and kidney (UA, UREA, and CREA) function indicators showing no abnormal changes. Histological analysis of major organs through hematoxylin and eosin (H&E) staining, as shown in fig. S11, further confirmed that the QDs did not induce any noticeable alterations in cell morphology. In addition, terminal deoxynucleotidyl transferase–mediated deoxyuridine triphosphate nick end labeling (TUNEL) staining revealed that the QDs did not lead to obvious apoptosis in major organs (fig. S12). In brief, all above experimental results demonstrate that the PEG-QDs exhibit excellent biocompatible profiles for in vivo applications.

### Optical imaging of TBI in vivo and 3D histopathological analysis

Based on the favorable optical and magnetic properties of ZCIS@ZnS:Mn QDs, as well as the excellent biocompatibility of the surface modification, the PEG-QDs were used for detecting TBI in vivo. To establish the TBI mouse model, a controlled free-fall impact was applied to one hemisphere of BALB/c nude mice. QDs were administered intravenously at a dose of 27 mg QDs per kg body weight for the following optical imaging, as schematically shown in Fig. 4A.

**Fig. 4.**
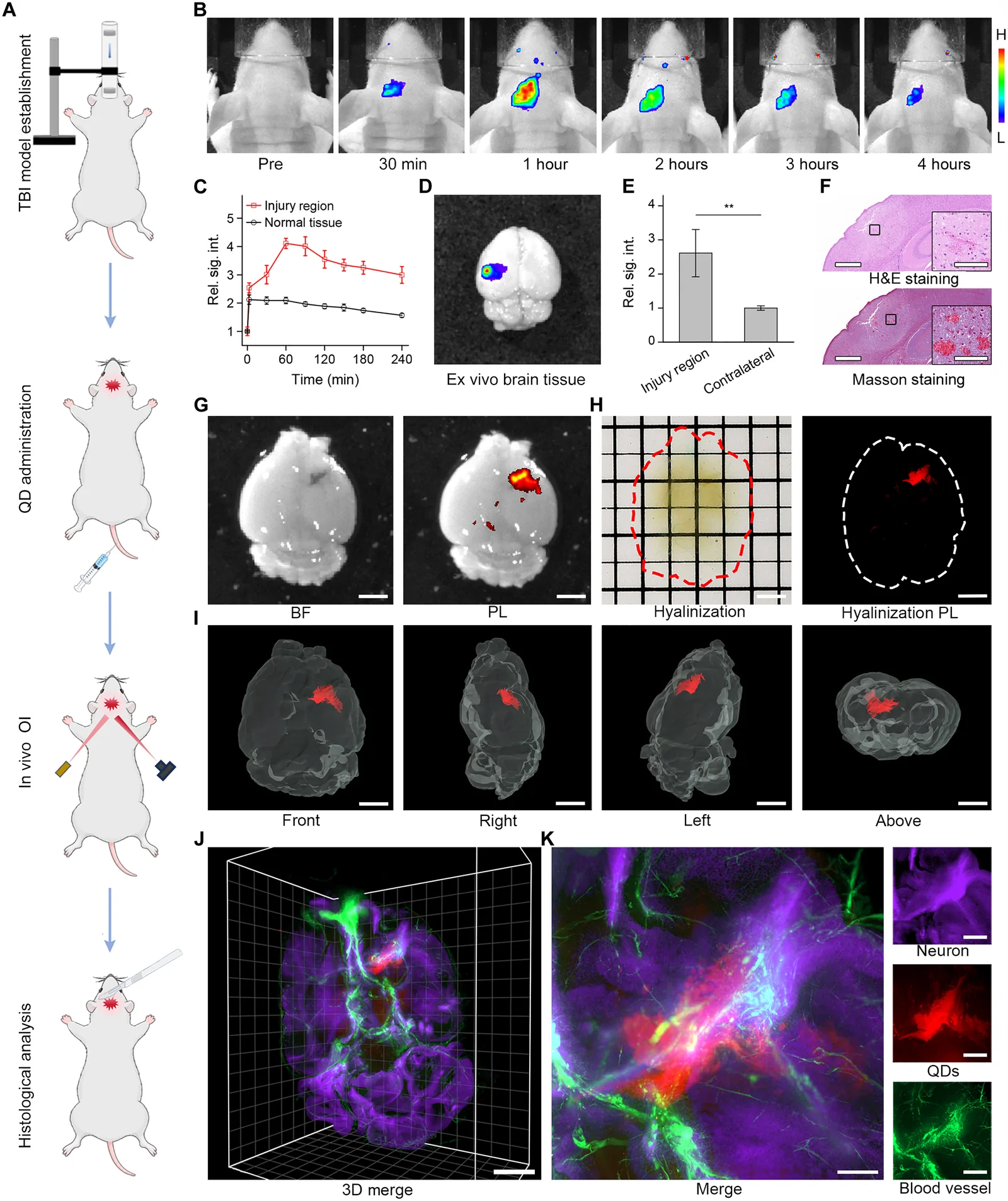
In vivo optical imaging of TBI with QDs, optical clearing, and 3D panoptic imaging of the TBI brain. (**A**) Schematic illustration of the procedure for the TBI model establishment and QD-based optical imaging. (**B**) In vivo NIR fluorescence images of TBI mouse recorded before and at different time points after intravenous injection of QDs, together with the (**C**) corresponding temporal evolution of fluorescence signals extracted from injured regions. (**D**) Ex vivo fluorescence images of brain tissues extracted from TBI mouse after imaging, together with (**E**) the corresponding relative signal intensity of injured region and contralateral normal region of brain. (**F**) Histological analysis of the brain slices. Scale bars, 500 μm (overview) and 100 μm (enlarged view). Ex vivo bright-field and fluorescence images of the TBI brain before (**G**) and (**H**) after the optical clearing process (scale bars, 3 mm). (**I**) Wide-field view of the entire cleared TBI brain (scale bars, 3 mm). (**J**) 3D panoptic immunofluorescence imaging of the entire cleared TBI brain (scale bar, 2 mm), with (**K**) an enlarged view of an injured region presented in multiple channels (purple: NeuN; green: CD31; red: QDs; scale bars, 0.5 mm for merge and 1 mm for single channels). Data were shown as mean ± SD. Statistical significance was determined by a *t* test (**P < 0.01). Panel (A) was produced by Microsoft Office 2021.

The mouse head was first imaged before and at different time points after intravenous injection of QDs through NIR fluorescence. As shown in Fig. 4B, the traumatic injury becomes detectable 30 min after QD injection, evidenced by a distinct fluorescence signal from the left side of the mouse’s head, coinciding well with the site of impact for establishing the TBI model. To quantify the fluorescence signal, the signal intensities from three different injured regions were extracted and averaged for comparing with those from the surrounding normal regions. As shown in Fig. 4C, the fluorescence signal is progressively increased against time to reach an intensity peak at ∼60 min postinjection, indicating a continuous accumulation of QDs in the injury site. Subsequently, the fluorescence intensity is gradually decreased over the following hours but remains apparently higher than that of the background.

To further validate the capabilities of the QDs for TBI detection, the TBI mouse was euthanized to extract the brain tissues for ex vivo imaging. As shown in Fig. 4D, a strong fluorescence signal remains clearly detectable in the left hemisphere of the brain, confirming the highly specific accumulation of QDs in the traumatic region with a relative signal intensity up to 2.6-fold higher than that of the corresponding contralateral region (Fig. 4E).

To provide a direct proof on that the fluorescence signal’s coming from the TBI site, histological analysis on brain tissue was performed. As shown in Fig. 4F, H&E staining well distinguishes the injured region in the brain cortex from the surrounding health tissues by noticeable morphological changes on both cell and tissue levels. For example, the injured regions exhibit weaker staining due to the disruption of tissue structure. In addition to H&E staining, Masson’s trichrome staining, as given in the bottom panel of Fig. 4F, clearly shows that multiple hemorrhagic foci appear in the TBI region, with red blood cells (RBCs) extravasating from blood vessels and accumulating within the brain parenchyma. Further Nissl staining was also employed to visualize the neuronal alterations caused by injury. As shown in fig. S13, neurons in the injured regions of the cortex appeared shallow in color in comparison to the surrounding area. These histological results strongly confirm that the fluorescence imaging comes from the TBI region in the brain cortex.

To gain deeper pathological insight into the QD-based imaging of TBI, advanced whole-brain clearing and imaging, i.e., CUBIC (clear, unobstructed brain imaging cocktails and computational analysis), was carried out. Following the optical clearing, the brain tissues become transparent, as shown in Fig. 4 (G and H), which is an important step for the following cell-resolved brain imaging. Through three-dimensional (3D) panoptic fluorescence imaging, as shown in Fig. 4I, it can be seen that the red fluorescence of QDs comes mainly from the traumatic injury region.

To pathologically understand the spatial distribution of the QDs’ signals, immunofluorescence analysis was performed by staining blood vessels and neurons through CD31 (green) and NeuN (neuronal nuclear antigen; purple), respectively. As illustrated in Fig. 4J and movie S1, 3D panoptic immunofluorescence imaging of the entire mouse brain confirms that the whole brain can roughly be delineated by the purple fluorescence, in which the QDs are stereotactically localized in the cerebral cortex adjacent to the right side of superior sagittal sinus, surrounded by CD31-positive green signals with a remarkable expansion feature, likely due to compensatory vasodilation of cortical vessels. To show the detailed distribution of the QDs within the injured region, high resolution imaging was performed. As shown in Fig. 4K and movie S1, the QD signals are predominantly localized around the dilated blood vessels, suggesting that QDs tend to extravasate through these vessels with enhanced permeability. To disclose the spatial distribution of QDs in more detail, four distinct imaging planes were selected from the 3D immunofluorescence LSFM dataset, and the fluorescence intensities were subsequently extracted through CD31 (blood vessels), NeuN (neuron), and QD channels, respectively. According to the results shown in Figs. 4K and 5 (A and B), the QDs form a complementary signal pattern with NeuN, whereas the green fluorescence from blood vessels passes continuously across the purple and red regions (Fig. 4K), indicating that the PEG-QDs are somehow specifically localized in the area of the degenerated neurons, showing a significant difference in fluorescence signal intensity between the injured and normal regions (*P* < 0.001), as given in Fig. 5C.

**Fig. 5.**
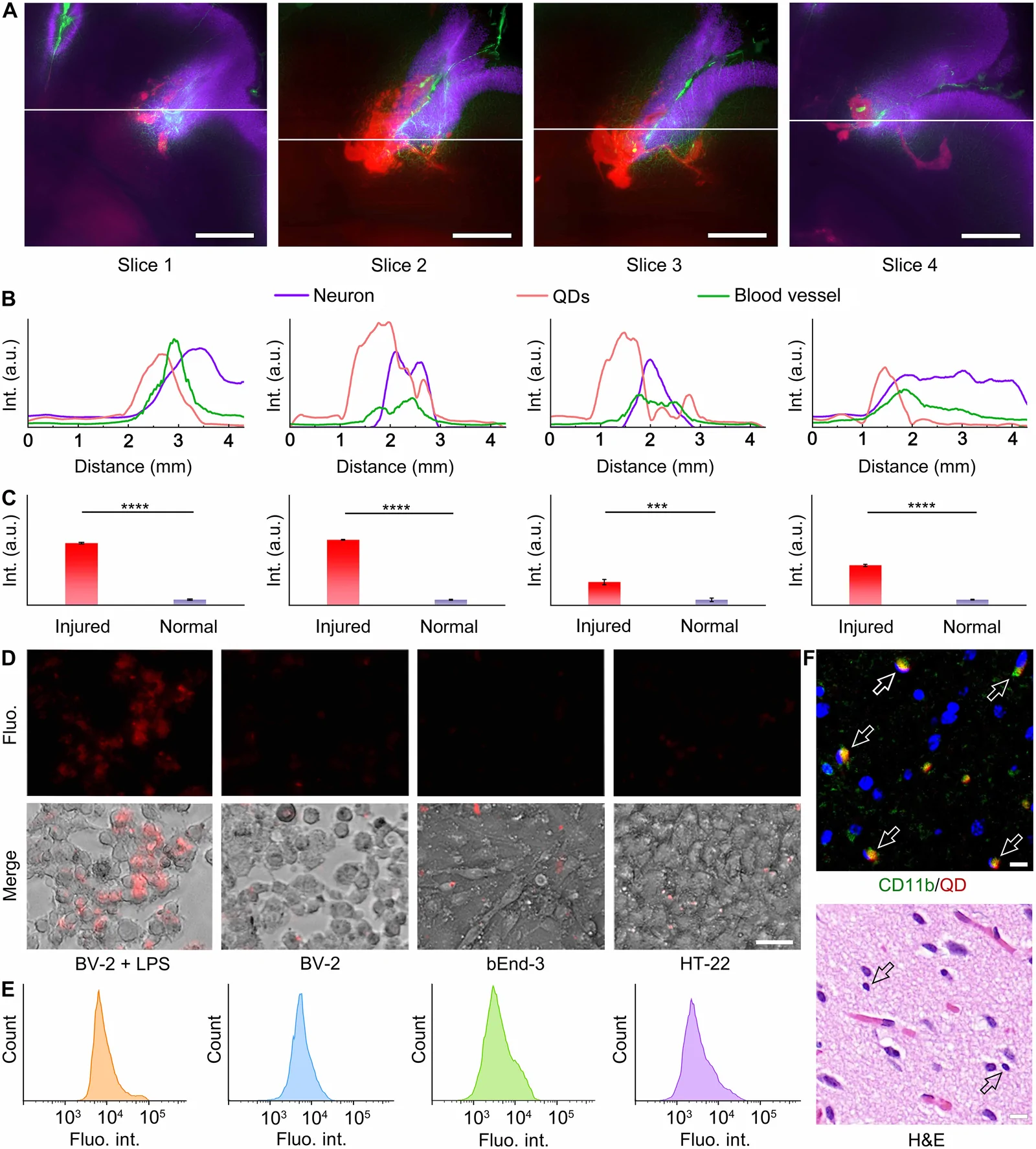
Mechanism of QD-based high-sensitivity imaging for TBI diagnosis. (**A**) Four representative imaging planes from the 3D panoptic immunofluorescence dataset of the injured brain region shown in Fig. 4K (scale bars, 1 mm), accompanied by line-scanning profiles across different channels (**B**) and a comparison of fluorescence signal intensities between injured and normal regions (**C**). (**D**) Fluorescence imaging of different cell types after coincubation with QDs. Scale bar, 50 μm. (**E**) Quantitative assessment of the cellular QD uptake by flow cytometry. (**F**) Top: Colocalization of QDs with CD11b-positive microglia in the injured brain region of TBI mice. Representative images show CD11b (green), QD (red), and DAPI (blue). Bottom: Corresponding H&E staining image of the brain injured region. Scale bar, 10 μm. Data were shown as mean ± SD. Statistical significance was determined by a *t* test (***P < 0.001 and ****P < 0.0001).

To understand the distribution pattern of QDs in vivo, microglia, a resident phagocyte of the central nervous system and key component of the mononuclear phagocyte system (MPS), came into focus. Following TBI, microglia rapidly migrate to accumulate in areas of hemorrhage and dead neurons, where they phagocytose cellular debris and regulate inflammatory responses and tissue repairing (*39*). It is therefore reasonable to hypothesize that QDs are specifically uptaken by activated microglia, leading to their selective accumulation in degenerated neuronal regions. To verify this hypothesis, the QD uptake capacity of different cell types, including neurons (HT-22), brain microvascular endothelial cells (bEnd-3), and microglia (BV-2), was evaluated. As shown in Fig. 5D, lipopolysaccharide (LPS)–activated BV-2 cells exhibit bright fluorescence after incubation with QDs. In contrast, the nonactivated BV-2 cells, bEnd-3 cells, and HT-22 cells present very weak fluorescence of QDs. This marked difference in QD uptake was further quantitatively confirmed by flow cytometric analysis (Fig. 5E). These results suggest that in the injury region, QDs are preferentially taken up by the activated microglia rather than the intact neurons or vascular endothelial cells.

To further confirm the preferential cellular uptake of the PEG-QDs by activated microglia, immunofluorescence staining was performed on brain sections that were extracted from QD-treated TBI mice, and anti-CD11b antibody was used for staining the activated microglia. As presented in Fig. 5F, prominent CD11b-positive signals (green) were observed in the injured brain region (indicated by white arrows). A large percentage of these CD11b-positive cells were simultaneously stained red, showing a high degree of colocalization with the green fluorescence. These results provided strong evidence on the accumulation of QDs in the activated microglia in the injured region.

### Advancing MRI of TBI toward a pathology-informed imaging

All above results validate the feasibility of NIR fluorescent QDs for TBI imaging in vivo. It is therefore interesting to know whether TBI can be diagnosed with more clinically biocompatible MRI through the same Mn-doped QDs. Following the intravenous injection of magnetic QDs, the brain MR images of TBI mice were recorded before and at various time points postinjection of the magnetic QDs. As shown in Fig. 6A, the TBI region can be identified in the cerebral cortex through *T*_1_-weighted imaging enhanced by the magnetic QDs. Quantitative analysis revealed that the contrast-to-noise ratio (CNR) and signal-to-noise ratio (SNR) of the injured region were improved by 4.5- and 1.2-fold (figs. S14 and S15), respectively, indicating excellent imaging contrast and sufficient signal quality for clear delineation of the brain trauma. To more precisely delineate the injury boundaries, 3D dynamic contrast enhanced (DCE) MRI sequence was applied. As indicated in Fig. 6B and movie S2, the injured region becomes distinctly visible upon enhancement by the QDs. In comparison to the conventional 2D imaging, the 3D imaging apparently provides more precise localization of the injury area. For instance, in the dorsal view of the initial impact site and the congestion region were measured to be 3.0 mm by 1.9 mm and 5.3 mm by 5.9 mm, respectively. From the side view, the thickness of the scalp hematoma was around 0.8 mm, and the depth of the traumatic injury was ∼1.7 mm from the scalp. Apparently, this 3D stereoscopic MRI provides more detailed delineation of the injury than fluorescence imaging although the latter is much more sensitive.

**Fig. 6.**
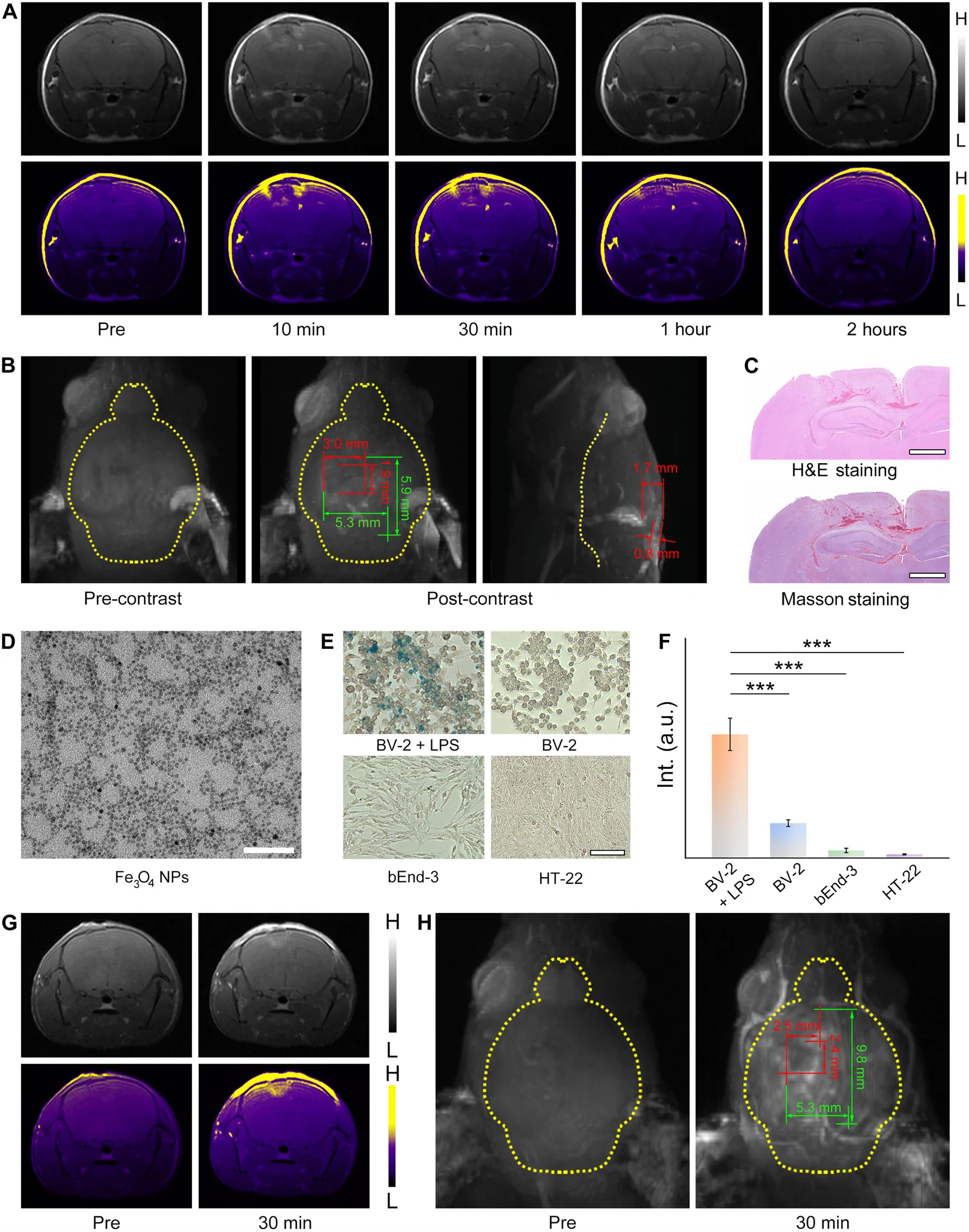
MRI of TBI based on magnetic QDs and PEGylated Fe_3_O_4_ nanoparticles, respectively. (**A**) In vivo *T*_1_-weighted MR images of TBI mouse head recorded before and at different time points after the intravenous injection of Mn-doped QDs. (**B**) 3D MR images of TBI mouse head recorded before and after the intravenous injection of QDs. (**C**) Histological analysis of the brain slices (scale bars, 1 mm). (**D**) TEM image of PEGylated Fe_3_O_4_ nanoparticles (NPs) (scale bar, 50 nm). (**E**) Prussian blue staining of different cell types after coincubation with Fe_3_O_4_ nanoparticles (scale bar, 100 μm), together with the relative signal analysis (**F**). (**G**) In vivo *T*_1_-weighted MR images and (**H**) 3D MR images of TBI mouse head recorded before and at 30 min after the intravenous injection of PEGylated Fe_3_O_4_ nanoparticles. Data were shown as mean ± SD. Statistical significance was determined by a one-way ANOVA (***P < 0.001).

To corroborate the diagnostic results obtained through MRI, the histological analysis on brain tissue were conducted. Adjacent slices were stained with H&E, Masson’s trichrome, and Nissl, respectively. As shown in Fig. 6C and fig. S16, the lesion boundaries identified histologically exhibit strong agreement with MRI results, confirming that the QD-based MRI is well suitable for showing the precise location of TBI.

According to diagnostic insight gained through optical imaging mentioned above, the PEG-QDs are extravasated through permeable BBBs of TBI mouse to enter the injured region where they are preferentially uptaken by the activated microglia. The activated microglia then migrate to the areas of neuronal death to phagocytose cellular debris, which enables the specific imaging of the pathological regions. This reasonable mechanism not only explains the selective accumulation of PEG-QDs but also highlights their potential for detecting microglia-mediated neuroinflammation and neuronal degeneration in TBI.

It is thus very interesting to know whether the diagnostic ability of the magnetic QDs can be expanded to other nanoprobe systems, e.g., PEGylated Fe_3_O_4_ nanoparticles that have recently been proven for clinical trial by China Food and Drug Administration (CFDA) as an MRI contrast agent. The PEGylated Fe_3_O_4_ nanoparticles were previously used to enhance the MRI of mouse brain infected by toxoplasma gondii (*40*). Although strongly enhanced signals were observed from the impaired BBB, the underlying mechanisms for the specific enhancement remain far from fully understood. To address this issue, PEGylated Fe_3_O_4_ nanoparticles of 3.2 nm (Fig. 6D and fig. S17) were adopted to disclose the specific interactions with different types of associated cells. The PEG modification endowed the nanoparticle a hydrodynamic size of ∼10 nm and nearly neutral surface (figs. S18 and S19), and the longitudinal relaxivity was determined to be 5.2 mM^−1^ s^−1^, as shown in fig. S20. After incubation with cells in vitro, Prussian blue staining was carried out. Intense staining was observed from the LPS-activated BV-2 cells, whereas nonactivated BV-2, bEnd-3, and HT-22 cells exhibited only weak staining (Fig. 6E and fig. S21). This significant difference (*P* < 0.001) in Fe_3_O_4_ uptake was further confirmed by the quantitative analysis of blue signals (Fig. 6F). In a similar way, PEGylated NaGdF_4_ nanoparticles (∼3 nm; fig. S22) were also examined, and chlorophosphonazo III staining revealed that LPS-activated BV-2 cells displayed a cyan color, whereas nonactivated BV-2 cells, along with bEnd-3 and HT-22 cells, appeared purple (fig. S23). These results confirmed that the preferential uptake of nanoparticles by the activated microglia was somehow governed by the particle surface coating, which encouraged us to carry out TBI imaging by using the PEGylated Fe_3_O_4_ nanoparticles as the MR contrast agent, because the latter is already very close to clinical applications.

The PEGylated Fe_3_O_4_ nanoparticles were then compared with the magnetic QDs developed herein in MRI detection of TBI. As shown in Fig. 6 (G and H) and figs. S24 and S25, the PEGylated Fe_3_O_4_ nanoparticle produced highly comparable patterns of TBI in both *T*_1_-weighted and 3D imaging in vivo, confirming that the cell-targeting behavior, primarily governed by extravasation of the imaging agents through the compromised BBB, followed by active uptake by recruited microglia, is universal for the PEGylated nanoparticles of similar sizes, irrespective of their core material. Therefore, it can be concluded that the detailed cellular and molecular insights gained from our QD-based optical studies provide a fundamental framework for advancing MRI of TBI from a nonspecific anatomical diagnosis toward a targeted, pathology-informed imaging with the aid of PEGylated magnetic nanoparticles.

A rigorous comparison between contrast-enhanced and nonenhanced MRI well highlights the value of the current studies. Nonenhanced *T*_1_-weighted imaging primarily reveals the gross anatomical disruption, whereas the enhanced MRI signals emerge specifically in areas of BBB breakdown and colocalize with subsequent dead neurons, as optically validated (Figs. 4 and 5). The evolvement of the imaging pattern, particularly in the peri-lesion area (Fig. 6), can reasonably be attributed to the active recruitment and accumulation of nanoprobe-laden microglia, which marks an important finding with the current dual-modality nanoreporter. In this context, the hyperintense regions on *T*_1_-weighted MR images no longer indicate the gross structural damage. They reflect specific, dynamic pathology, including focal BBB leakage, and the subsequent migration and accumulation of the activated microglia. Those are the key follow-up of TBI, as evidenced by the spatiotemporal evolution of the MRI signal intensity in the peri-lesion area (Fig. 6), far beyond the scope of conventional MRI.

In summary, we herein move from simply using nanomaterials as contrast agents to making them targeted reporters to gain pathological information through imaging. The mechanistic understanding gained herein has demonstrated that the nanoparticle-enhanced MRI can pinpoint the pathological signatures including BBB breakdown and neuroinflammatory responses, which marks a substantial leap over traditional anatomical MRI. Thus, this work represents a monentous advancement in neuroimaging, particularly for TBI, which is a leading cause of global mortality and disability in both civilian and military medicine worldwide.

## MATERIALS AND METHODS

### Experimental design

This study was designed to evaluate the diagnostic utility of Mn-doped NIR QDs in TBI, encompassing their capacity to integrate optical and MR imaging for pathological interpretation via microglia-mediated tropism, alongside biosafety, and comparative translational assessments with Fe_3_O_4_ nanoparticles. In vivo studies were generally preferred to evaluate the properties of nanoparticles, and animals were randomly assigned to different experimental groups at the onset of each study. Healthy mice, together with the TBI models were adopted to evaluate the properties of QDs in distinct aspects. In the biosafety evaluation experiments, researchers who performed data collection and analysis were blinded to group allocation and detailed materials information, and in in vivo imaging studies, researchers who performed data collection and analysis were blinded to group allocation and detailed animals’ information. In vitro studies and in vivo studies were performed with at least three replicates, unless otherwise noted. No data were excluded from the experiments. All animal experiments were approved by the Animal Ethics Committee of Qingdao University of Science and Technology Laboratory (no. QKDLL-2026-38) and reported in accordance with the ARRIVE guidelines 2.0.

### Chemicals

Indium(III) chloride tetrahydrate (InCl_3_·4H_2_O; 97%) was purchased from Adamas-beta. Copper(II) chloride dihydrate (CuCl_2_·2H_2_O; ≥99.0%), sodium citrate dihydrate (≥99.0%), manganese(II) chloride tetrahydrate (MnCl_2_·4H_2_O; 99%), GSH (≥98.0%), gadolinium(III) chloride hexahydrate (GdCl_3_·6H_2_O; 99.99%), 1-octadecene (90%), oleic acid (OA; 90%), and ammonium fluoride (NH_4_F; ≥98.0%) were purchased from Sigma-Aldrich. Zinc chloride (ZnCl_2_, 99.999% metals basis), NaOH (≥ 96%), and sodium sulfide nonahydrate (Na_2_S·9H_2_O, ≥ 99.99% metals basis) were purchased from Aladdin. Anhydrous ethanol (AR), toluene (AR), and sodium borohydride (NaBH_4_; 98%) were purchased from Sinopharm Chemical Reagent Co. Ltd. CCK-8, dimethyl sulfoxide (DMSO), Dulbecco’s modified Eagle’s medium (DMEM), and FBS were purchased from Sangon Biotech Co. Ltd. (Shanghai, China). Trypsin was purchased from Biosharp (Hefei, China). All chemicals and reagents were used as received without any further purification. Millipore ultrapure water (resistivity of 18.2 MΩ·cm) was used in all experiments. Asymmetric PEG [molecular weight (MW): 2000] was sourced from Beijing Oneder Hightech Co. Ltd. The PEGylated Fe_3_O_4_ nanoparticles used in this work were provided by Suzhou XinYing Biomedical Co. Ltd. (Suzhou, China).

### Synthesis of Mn-doped ZCIS@ZnS QDs

Typically, for preparing ZCIS QDs, CuCl_2_·2H_2_O (0.00625 mmol), ZnCl_2_ (0.00625 mmol), InCl_3_·4H_2_O (0.0375 mmol), Na_2_S (0.060 mmol), sodium citrate (0.150 mmol), and GSH (0.125 mmol) were added in sequence into 20 mL of deoxygenated ultrapure water in a 50-mL three-necked flask under magnetic stirring, yielding a transparent solution (pH ∼ 6). ZCIS core QDs were then obtained after refluxing the above solution for 2 hours.

For achieving Mn-doped ZCIS@ZnS QDs, Mn^2+^ ions were introduced in the ZnS shell. Specifically, a stock solution containing Zn and Mn precursors was first prepared by adding ZnCl_2_ (0.05 mmol), MnCl_2_ (0.0429 mmol), and GSH (0.05 mmol) to 2 mL of ultrapure water with pH value adjusted to 4 to 5 by using 0.006 mmol of NaOH. After the QD core reaction solution was cooled to 80°C, the Zn/Mn stock solution was introduced into the core QD solution to initiate the shell growing process. After reacting at 80°C for 2 hours, Mn-doped QDs were obtained. To vary the Mn doping level, the Mn precursor feed amount was changed accordingly, and the doping level was calculated as follows: Mn content (mol) divided by the total content of all cations including Zn, Cu, In, and Mn.

To purify the final product, fivefold of ethanol was added into the cooled reaction solution to precipitate the QDs, which was then centrifuged at 4000 rpm for 10 min to remove the supernatant. Afterward, the QD precipitation was redispersed in water. After three cycles of the above procedures, the final purified QD solution was stored at 4°C for further use.

### Surface biocompatible modification of QDs

To improve the biocompatibility of the QDs obtained, they were mixed with mPEG-SH by an mPEG:QDs ratio of 10:1 (mass) in aqueous solution, which was then shaken overnight to complete ligand exchange process. The excessive mPEG-SH was removed through ultrafiltration by a 30-kDa ultrafiltration tube for three cycles. The obtained PEG modified QDs were stored at 4°C for further use.

### Synthesis of PEGylated NaGdF_4_ nanoparticles

GdCl_3_·6H_2_O (1 mmol) was mixed with OA (4 mL) and 1-octadecene (15 mL) in a 100-mL flask. The mixture was heated at 150°C under vacuum for 30 min and then cool to 50°C. A methanolic solution (10 mL) containing NaOH (2.5 mmol) and NH_4_F (4 mmol) was added dropwise to the flask. After stirring overnight at 50°C, the system was heated to 100°C for 10 min under vacuum to evaporate methanol. Subsequently, the temperature was raised to 270°C quickly under nitrogen and maintained for 40 min. The resulting product was cooled to room temperature, washed several times with ethanol via centrifugation, and lastly dispersed in cyclohexane. To obtain PEGylated NaGdF_4_ nanoparticles, OA-capped NaGdF_4_ nanoparticles (10 mg) and mPEG-maleimide (100 mg) were dissolved in tetrahydrofuran (THF; 10 mL) and stirred at 40°C overnight. The obtained PEGylated nanoparticles were then precipitated using cyclohexane, collected by repeated centrifugation, and dried under vacuum at room temperature. Further purification was performed by ultrafiltration using a 30-kDa MWCO centrifugal filter to remove unbound ligands.

### Characterization

Steady-state ultraviolet-visible (UV-Vis) absorption spectra were recorded on a Shimadzu UV-2600 UV-Vis spectrophotometer. Steady-state PL spectra were recorded by an FLS1000 fluorescence spectrometer (with NIRPMT R5509 detector, Edinburgh Instruments, UK) under excitation at 500 nm. The time-resolved spectra (with a picosecond diode laser EPL-445) as well as the absolute PLQYs of QDs were also measured by an FLS1000 fluorescence spectrometer. To evaluate the stability of Mn-doped QDs, they were dispersed in ultrapure water, 1× PBS, and 10% FBS, respectively, for monitoring the changes of PL intensity. TEM and HRTEM studies were carried out using a JEM-2100F at 200 kV. The particle size was determined by averaging at least 300 particles per sample by ImageJ. EPR spectra were collected at room temperature on a Bruker EMX PLUS spectrometer. The chemical composition of purified QDs was determined by an inductively coupled plasma optical emission spectrometer using a Thermo Fisher Scientific IRIS Intrepid II XSP. XPS measurements were carried out on a Thermo Fisher Scientific ESCALAB 250Xi spectrometer with a monochromatic Al Kα x-ray source. Powder x-ray diffraction (PXRD) patterns of the samples were recorded on a Rigaku D/Max-2500 diffractometer under Cu Kα1 radiation (λ = 1.54056 Å). FTIR spectra were recorded on a Nicolet iS10 spectrometer (Thermo Fisher Scientific) within the range of 4000∼400 cm^−1^ to characterize the QDs with difference surface capping ligands. Hydrodynamic size and zeta potential were determined at 25°C using a Malvern Zetasizer Nano ZS, configured with a 632.8-nm He-Ne laser.

### Relaxivity measurements 

Relaxivity measurements were performed on a 3.0-T MRI scanner. Specifically, a set of QD solutions with varied concentrations was prepared. A range of distinct MRI sequences were adopted to assess the MRI performance of the Mn-doped QDs. The parameters of *T*_1_-weighted imaging were set as follows: repetition time (TR) = 720 ms, echo time (TE) = 11 ms, and field of view (FOV) = 40 by 40 mm; the parameters of *T*_2_-weighted imaging were set as follows: TR = 5000 ms, TE = 68 ms, and FOV = 40 by 40 mm.

### Cytotoxicity assessment

The cytotoxicity of the PEG-QDs was evaluated through CCK-8 assays across multiple cell lines representing different cell types, e.g., fibroblasts (NIH/3T3; Servicebio, catalog no. STCC20005G), brain microvascular endothelial cells (bEnd-3; Servicebio, catalog no. STCC20030G), microglia (BV-2; Servicebio, catalog no. STCC20009G), and neurons (HT-22; Servicebio, catalog no. STCC20011G). All cell lines were routinely tested and confirmed negative for mycoplasma contamination.

Typically, the cells were seeded into a 96-well plate with a cell density of 1 × 10^4^ cells per well and then incubated in a medium of DMEM supplemented with 10% FBS and 1% penicillin-streptomycin solution (100×) at 37°C in a 5% CO_2_ atmosphere for 24 hours. Afterward, the cells were washed twice by using 1× PBS and incubated with different concentrations of QDs at 37°C for another 24 hours. Next, the cells were rinsed three times using 1× PBS to eliminate free QDs. After that, they were placed in 100 μL of culture medium supplemented with 10 μL of CCK-8 and then incubated at 37°C for 1 hour. Last, the absorbance at 450 nm was measured on a microplate reader (TECAN, SPARK 10M).

### Hemolysis assay

For evaluating the toxicity of PEG-QDs in blood, the rupture of RBCs was detected after coincubated with QDs. Fresh rabbit blood (Henan Megalithic Biotechnology Co. Ltd., China), treated with EDTA (purchased from Henan Megalithic Biotechnology Co. Ltd., China) as an anticoagulant, was washed with 1× PBS and centrifuged at 1500 rpm for 5 min to obtain RBCs. Next, QDs of different concentrations in 1× PBS dispersions were mixed with RBCs and incubated at room temperature for 1 hour. Ultrapure water and 1× PBS were also mixed with RBCs, respectively, as controls. After incubation, the mixture was centrifuged at 1500 rpm for 10 min and the supernatant was transferred to a 96-well plate. The absorbance at 450 nm were measured on a microplate reader (TECAN, SPARK 10M).

### Cell imaging

To investigate the surface-dependent phagocytosis of QDs by macrophages (RAW264.7 cells), GSH-QDs and PEG-QDs were compared. Typically, the cells were seeded into a culture dish at a density of 6 × 10^4^ cells per dish and cultured at 37°C and a 5% CO_2_ atmosphere for 24 hours. After being washed three times with 1× PBS, the cells were incubated with QDs of 9 μM for 2 hours. After the cells were washed three times with 1× PBS to remove the free particles, the fluorescence signals of the cells at a 593-nm wavelength were recorded by using a fluorescence microscope (BioTek Cytation 5 imaging reader, USA), with an excitation wavelength of 469 nm.

To evaluate the ROS generation induced by the magnetic QDs, bEnd-3 cells were seeded in cell culture dishes and cultured at a density of 6 × 10^4^ cells per dish. After being washed with 1× PBS, the cells were incubated with QDs of different concentrations for 4 hours. Thereafter, the cells were rinsed again with 1× PBS buffer, and then the ROS detection Kit was used to display the ROS generation. The fluorescence imaging of cells was observed on a confocal microscope (BioTek Cytation 5 imaging reader, USA).

### Cell uptake assay

LPS-activated BV-2 cells, along with nonactivated BV-2, bEnd-3, and HT-22 cells, were seeded in 24-well plates at a density of 6 × 10^4^ cells per well and cultured for 24 hours. Cells were then incubated with QDs (9 μM) for 4 hours. After two washes with 1× PBS, cellular fluorescence was visualized under a fluorescence microscope and quantitatively analyzed by flow cytometry.

To evaluate the uptake of PEGylated Fe_3_O_4_ and NaGdF_4_ nanoparticles in parallel, the same four cell types were treated with PEGylated nanoparticles using the same protocol (the cell incubation concentrations of Fe_3_O_4_ and NaGdF_4_ nanoparticles were 160 μg/mL with respect to Fe and 80 μg/mL with respect to Gd, respectively). Cellular internalization was subsequently assessed via Prussian blue staining or chlorophosphonazo III staining.

### Animals

Male BALB/c mice and BALB/c nude mice (6 to 8 weeks old) were obtained from Jinan Pengyue Experimental Animal Breeding Co. Ltd. (China). All animals were housed in a specific pathogen–free (SPF) facility under a controlled environment (temperature: 22° ± 1°C; humidity: 50 to 60%) with a 12-hour light/12-hour dark cycle. The mice were maintained in groups of four per cage and provided with ad libitum access to standard rodent chow and autoclaved water.

### Biocompatibility assessment in vivo

Eight healthy BALB/c mice were randomly divided into two groups (*n* = 4): One group was injected with 1× PBS, and the other group was injected with PEG-QDs (containing 27 mg QDs per kg body weight). The weight of mice was monitored for 20 days, and then the mice were euthanized. The blood collected from mice was further used for blood routine and biochemical testing. The main organs of mice, including the liver, spleen, kidney, heart, and lungs, were collected and fixed with 10% neutral buffered formalin, embedded in paraffin and then sliced to thin sections. H&E and TUNEL staining were carried out for histological analysis. The fluorescence of slice was observed with a fluorescence microscope (BioTek CYTATION 5 imaging reader, USA).

### TBI mouse model

Weight-drop TBI mouse model was used to assess the feasibility of the magnetic QDs in TBI diagnosis. Brain injury was caused by free-fall movement. First, mice were anesthetized with isoflurane and fixed on the stereotactic device. Next, the head of mice were shaved and disinfected carefully. Then, a 20-g weight was dropped from 15 cm above the head of each mouse to strike the dura mater vertically, resulting in TBI.

### Optical imaging of TBI in vivo

First, the TBI mice were anesthetized with isoflurane and placed on a small animal imaging system (PerkinElmer IVIS Lumina series III, USA). PEG-QDs dispersed in 1× PBS were intravenously injected through the tail vein at a dosage of 27 mg QDs per kg body weight. Temporal fluorescence from TBI brain were recorded at different time points postinjection. The excitation and emission wavelength were set as 600 and 710 nm, respectively, with built-in filters, and the exposure time was set as 8 s. Healthy mice were used as control group under the same imaging conditions. The fluorescence images were analyzed using the AniView software. At specific imaging time points, mice were euthanized, and their brains were dissected, and adjacent brain sections were treated with H&E, Masson’s trichrome staining, and Nissl staining, respectively. Immunofluorescence staining was also conducted on brain sections using anti-CD11b (Servicebio, catalog no. GB15058) antibody, followed by incubation with fluorescent secondary antibody and 4′,6-diamidino-2-phenylindole (DAPI) counterstaining.

### In vivo MRI

Brain MRI was acquired on a 7.0-T Bruker Biospec animal MRI scanner equipped with a mouse head surface coil. *T*_1_-weighted imaging and DCE MR sequences were adopted. The detailed parameters for these sequences are as follows: *T*_1_-weighted imaging: TR = 500 ms, TE = 5.58 ms, FOV = 25 by 25 mm, matrix = 200 by 200, and slice thickness = 0.8 mm; 3D DCE: TE = 1.41 ms, TR = 14.01 ms, FOV = 18 mm by 17 mm, MTX = 138 by 131, and slice thickness = 0.3 mm. During MRI experiments, mice were anesthetized with 2% isoflurane in oxygen-mixed air via a face mask before and after intravenous injection of QDs (27 mg per kg body weight) or PEGylated Fe_3_O_4_ nanoparticles (10 mg per kg body weight).

### 3D brain tissue imaging and image processing

Brain tissue clearing was carried out by using a tissue clearing kit (Nuohai Life Science Co. Ltd.) to eliminate interference of spontaneous fluorescence background of the brain tissue.

Immunofluorescence staining was conducted to label neurons and vascular through NeuN and CD31, respectively, following the fluorescence imaging of the brain with the QDs (anti-NeuN antibody, Abcam, catalog no. ab177487; anti-CD31 antibody, R&D Systems, catalog no. AF3628). 3D fluorescence imaging of the dyed brain tissues was conducted with a Nuohai LS 18 Tiling Light Sheet Microscope (Nuohai Life Science Co. Ltd., laser lines: 488 nm for NeuN, 561 nm for QDs, and 637 nm for CD31). A four-tile tiling light sheet was used to illuminate the sample, and a 1×/0.25–numerical aperture objective (Olympus MVPLAPO) was used to collect the fluorescence. The magnification of the microscope was set as 4×, and the spatial resolution was roughly 3.3 μm by 3.3 μm by 7 μm at the selected imaging conditions.

The fluorescence images were processed with the LS 18 Image Combine software [Nuohai Life Science (Shanghai) Co. Ltd.] and rendered using Amira 2021.2 (Thermo Fisher Scientific, USA). The 3D modeling process in Amira 2021.2 (Thermo Fisher Scientific, USA) followed a streamlined pipeline that transforms raw imaging data into a quantifiable digital model. It began with the import of a 3D image stack, which was preprocessed using filters to enhance quality and reduce noise. The core step was segmentation, where the structure of interest was precisely defined using thresholding and manual labeling. This labeled data were then converted into a smooth, polygonal surface mesh using the surface generation module. Last, the model was established for 3D representation.

### Statistical analysis

Quantitative data are expressed as mean ± SD. The comparisons between two groups were evaluated using Student’s *t* test, and comparisons among multiple groups were assessed by one way analysis of variance (ANOVA), with *P* < 0.05 considered statistically significant.
