# Supplementary material for: Lighting up necrotic neurons from traumatic brain injury with magnetic quantum dots to decode the cellular origin of MRI
Source: Sci Adv. 2026 Nov 4;12(45):eaej0686. doi: 10.1126/sciadv.aej0686 (PMC13733891; doi:10.1126/sciadv.aej0686)
Supplement: Supplementary file 1 [file sciadv.aej0686_sm.pdf]

Science Advances
AAAS

Supplementary Materials for

Lighting up necrotic neurons from traumatic brain injury with magnetic quantum dots to decode the cellular origin of MRI

Mingxia Jiao *et al.*

Corresponding author: Xiliang Luo, xiliangluo@qust.edu.cn; Peisen Zhang, zhangps@qust.edu.cn; Mingyuan Gao, gaomy@suda.edu.cn

Sci. Adv. **12**, eaej0686 (2026)
DOI: 10.1126/sciadv.aej0686

The PDF file includes:

Supplementary Text
Figs. S1 to S25
Tables S1 to S3
Legends for movies S1 and S2

Other Supplementary Material for this manuscript includes the following:

Movies S1 and S2

Supplementary Text

Time-resolved photoluminescence (TR PL) measurements

The PL decay curves were fit using the multi-exponential function:

$$I(t) = \sum_{i=1}^{n} B_i \exp(-t/\tau_i), \quad \sum_{i=1}^{n} B_i = 1 \qquad S(1)$$

In this expression, τ_i represents the decay time constants, B_i represents the normalized amplitudes of each component, and *n* is the number of decay times. Because the photoluminescence decays for all the QDs are best fit using a three-exponential function ($n = 3$), the amplitude-weighted average decay lifetime τ_{avg} of the entire fluorescence decay process was calculated in the form:

$$\tau_{avg} = \frac{\sum B_i \tau_i^2}{\sum B_i \tau_i} \qquad S(2)$$

The normalized lifetime-amplitude product is given as:

$$f_i = \frac{\tau_i B_i}{\sum \tau_i B_i} \qquad S(3)$$

In this expression, f_i represents the relative time-integrated contribution of each respective process to the overall number of emitted photons (i.e., the emission intensity measured in steady state PL spectra).

The radiative and nonradiative recombination rates with the PLQY were further correlated to understand the fluorescence behavior of the QDs with different Mn doping levels. The radiative recombination rate (τ_r^{-1}) can be determined by the experimentally determined PLQY in **Fig. 1a** and the measure average PL lifetime (τ_{avg}) in **Table S2** as follows:

$$\tau_r^{-1} = QY \times \tau_{avg}^{-1} \qquad S(4)$$

and then the non-radiative recombination rate (τ_{nr}^{-1}) was calculated assuming no dark fraction of emitters and is given as:

$$\tau_{nr}^{-1} = \tau_{avg}^{-1} - \tau_r^{-1} \qquad S(5)$$

The PL decay-fitting data for all curves are summarized in **Table S2-S3**.

Supplementary Figures

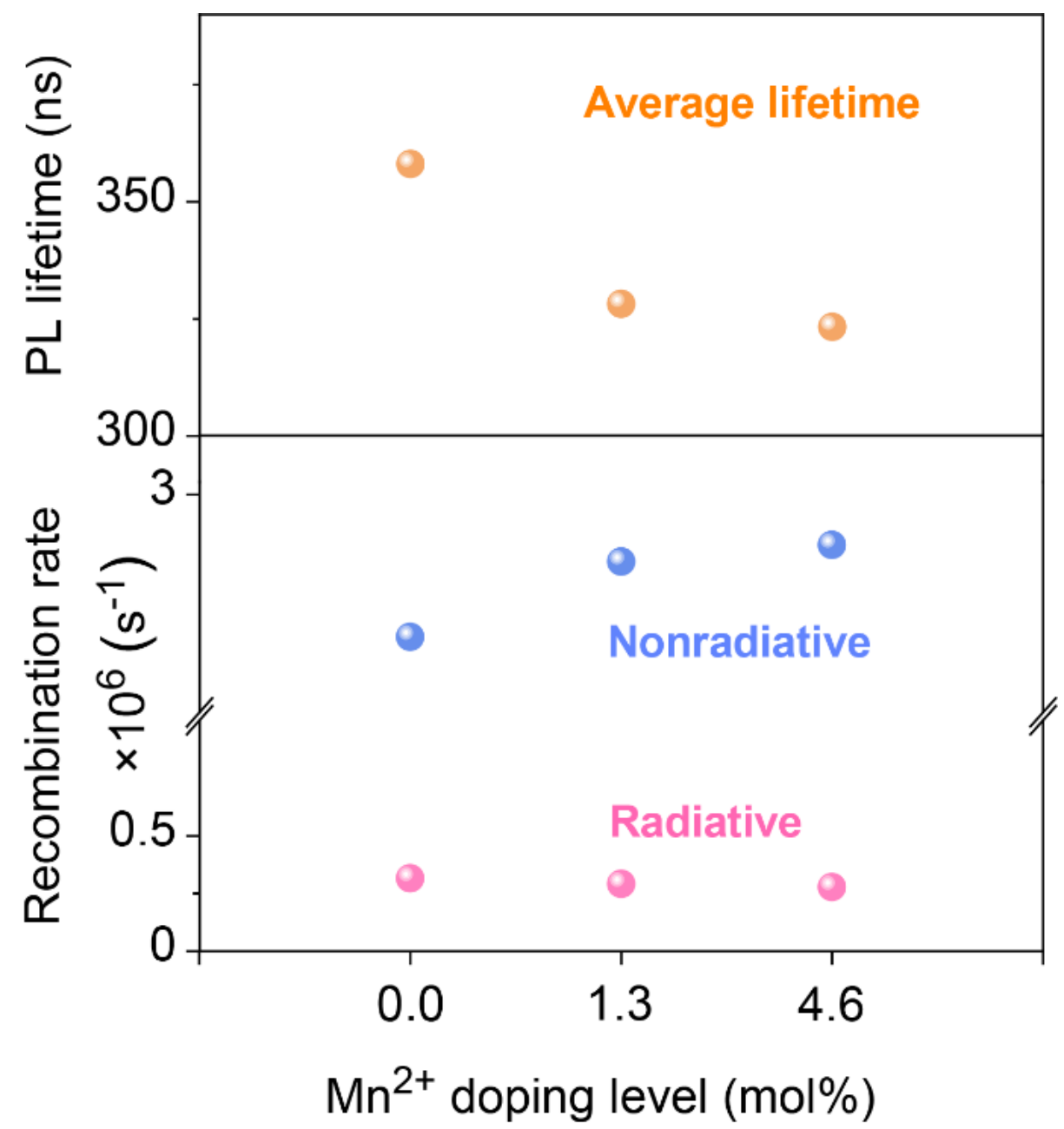

Fig. S1.

Transient optical properties of Mn-doped QDs. Average lifetime and nonradiative/radiative recombination rates of magnetic QDs as a function of Mn-doping levels.

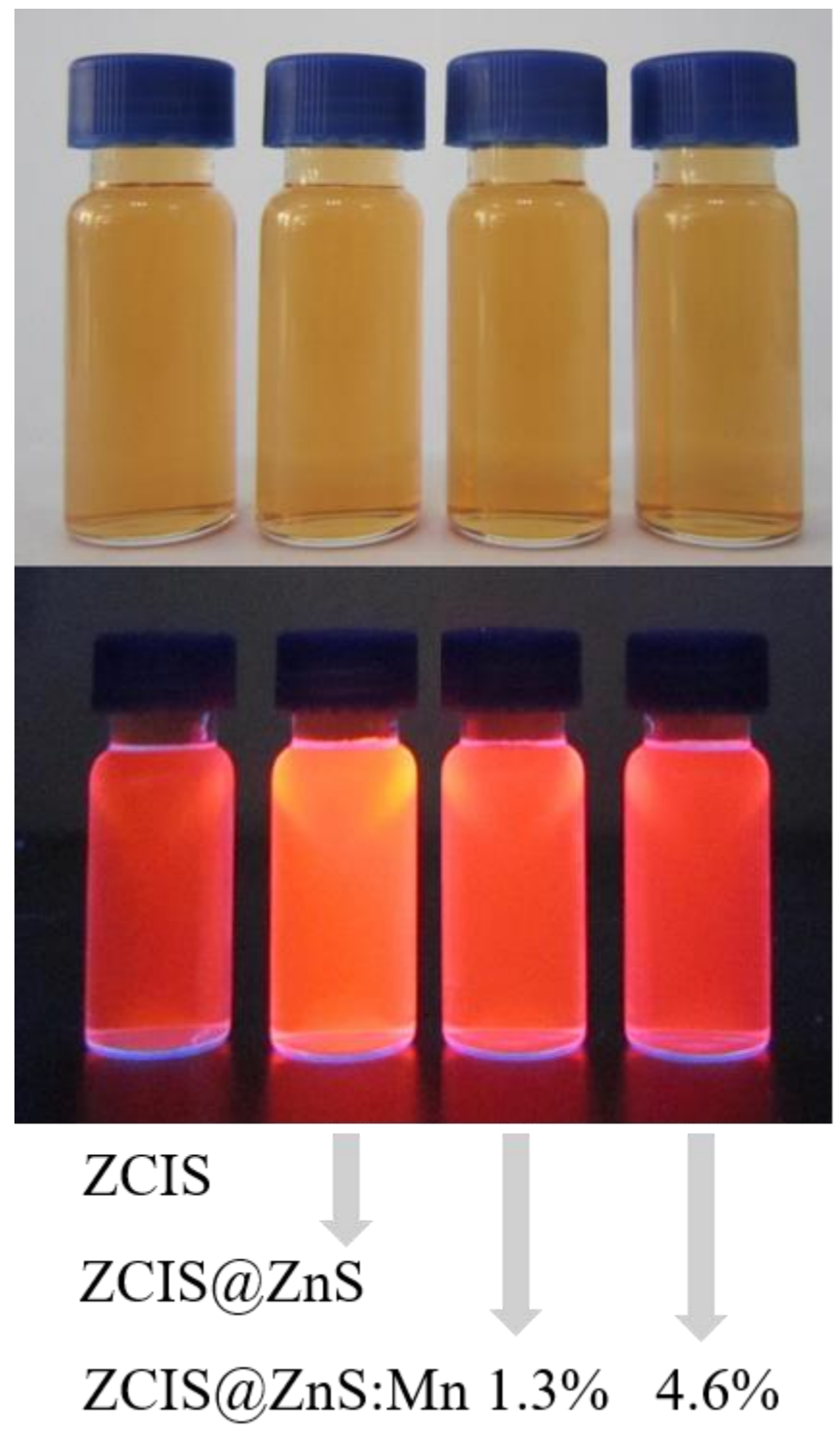

Fig. S2.

Visual comparison of the fluorescence of QDs. Photographs of the QDs dispersed in water under room light and UV excitation.

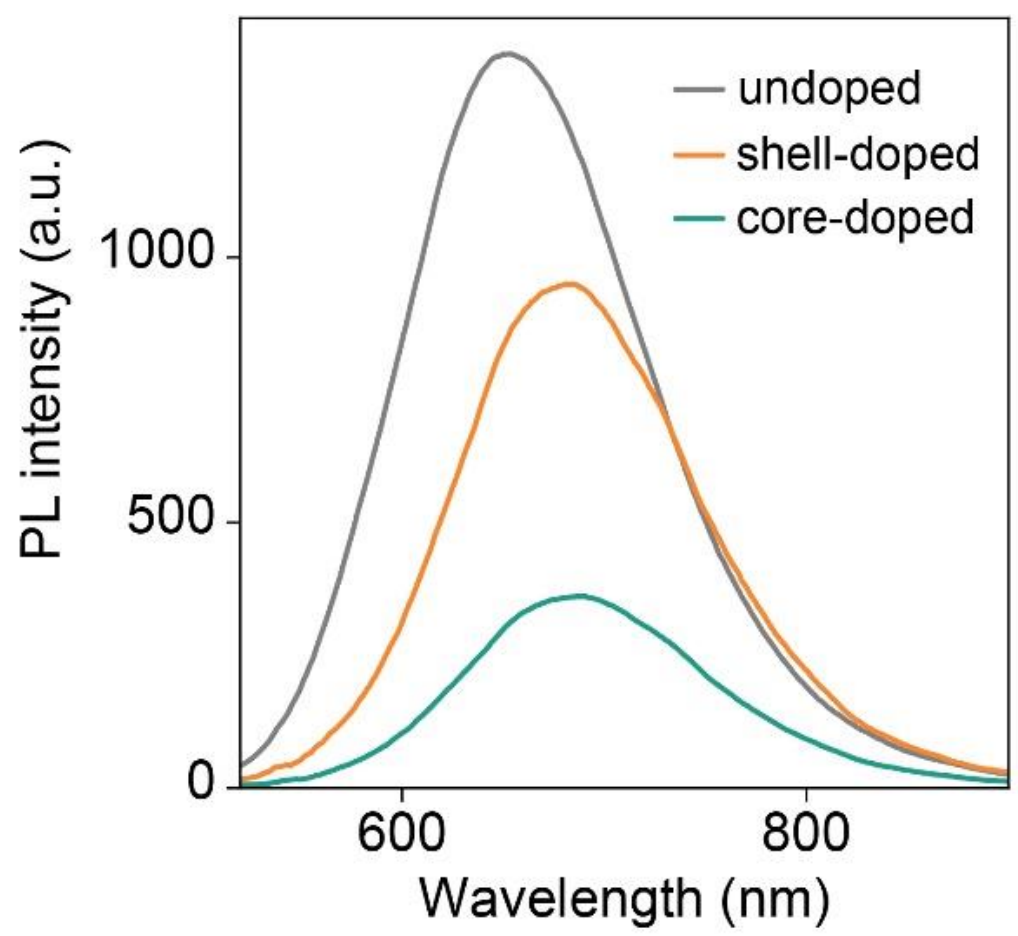

Fig. S3.

Influence of Mn-doping on the fluorescence of QDs. PL spectra of undoped and Mn-doped QDs through different doping strategies (Mn-doping level: 4.6%).

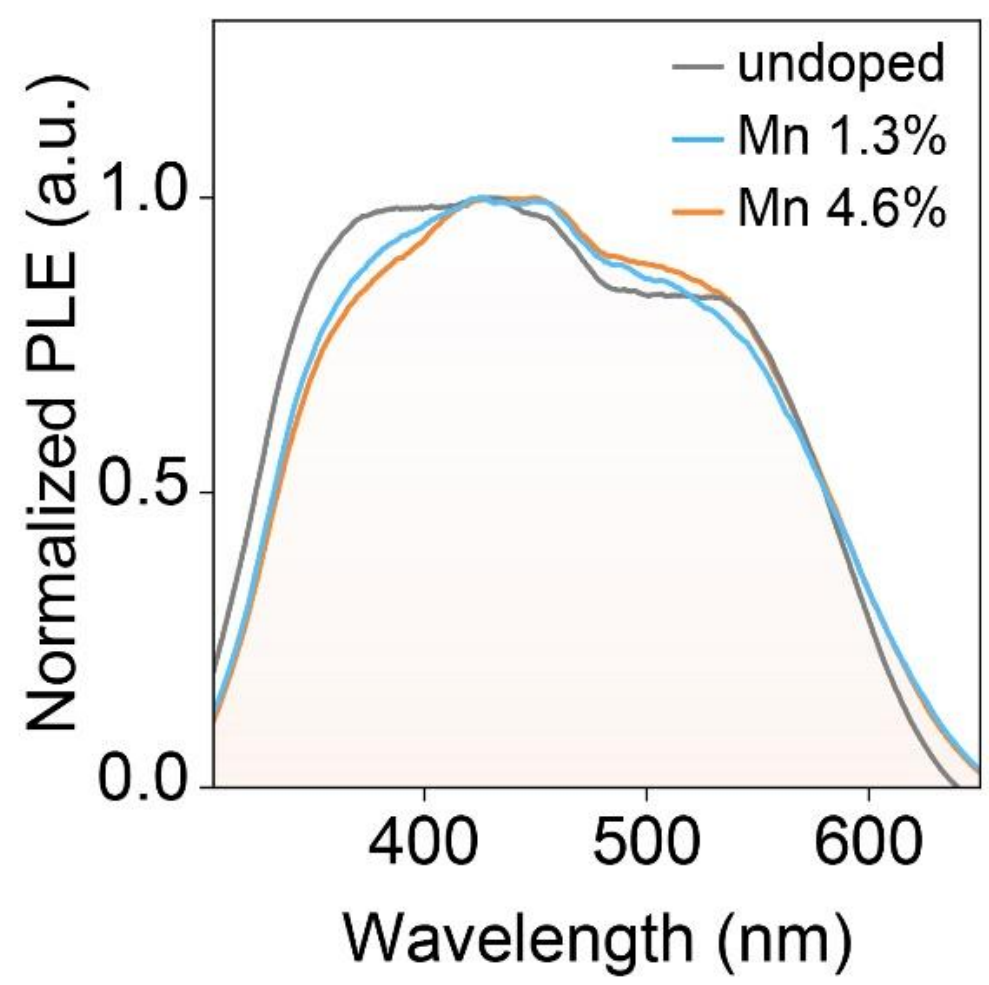

Fig. S4.

Broad excitation range of Mn-doped QDs. PL excitation spectra of QDs with different Mn-doping levels.

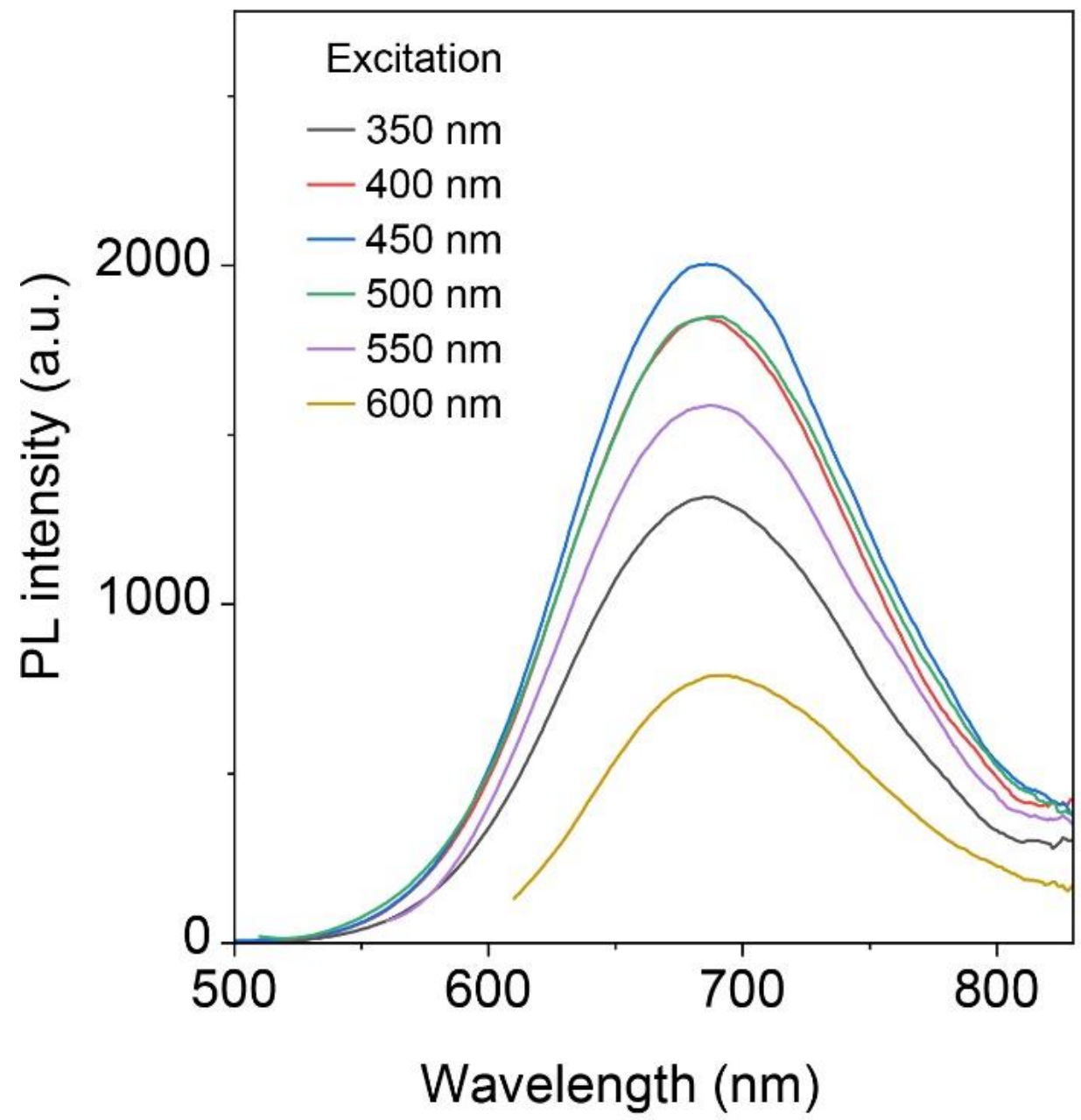

Fig. S5.

Excitation-independent emission property of Mn-doped QDs. PL spectra of QDs with Mn-doping level of 4.6% at different excitation wavelengths.

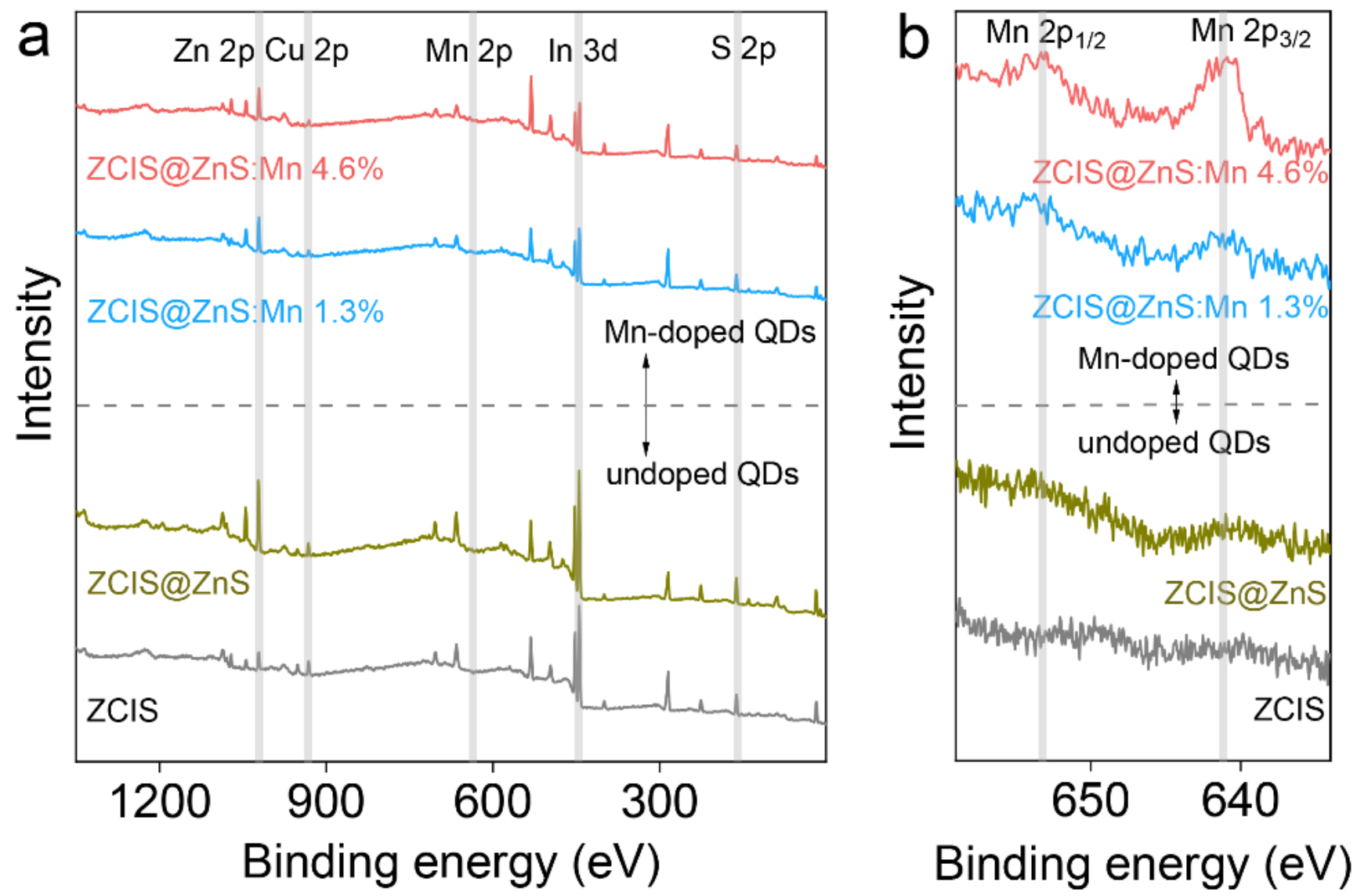

Fig. S6.

Gradual change of Mn content for QDs with different doping levels. XPS spectra (a) and high-resolution XPS spectra (b) of different QDs.

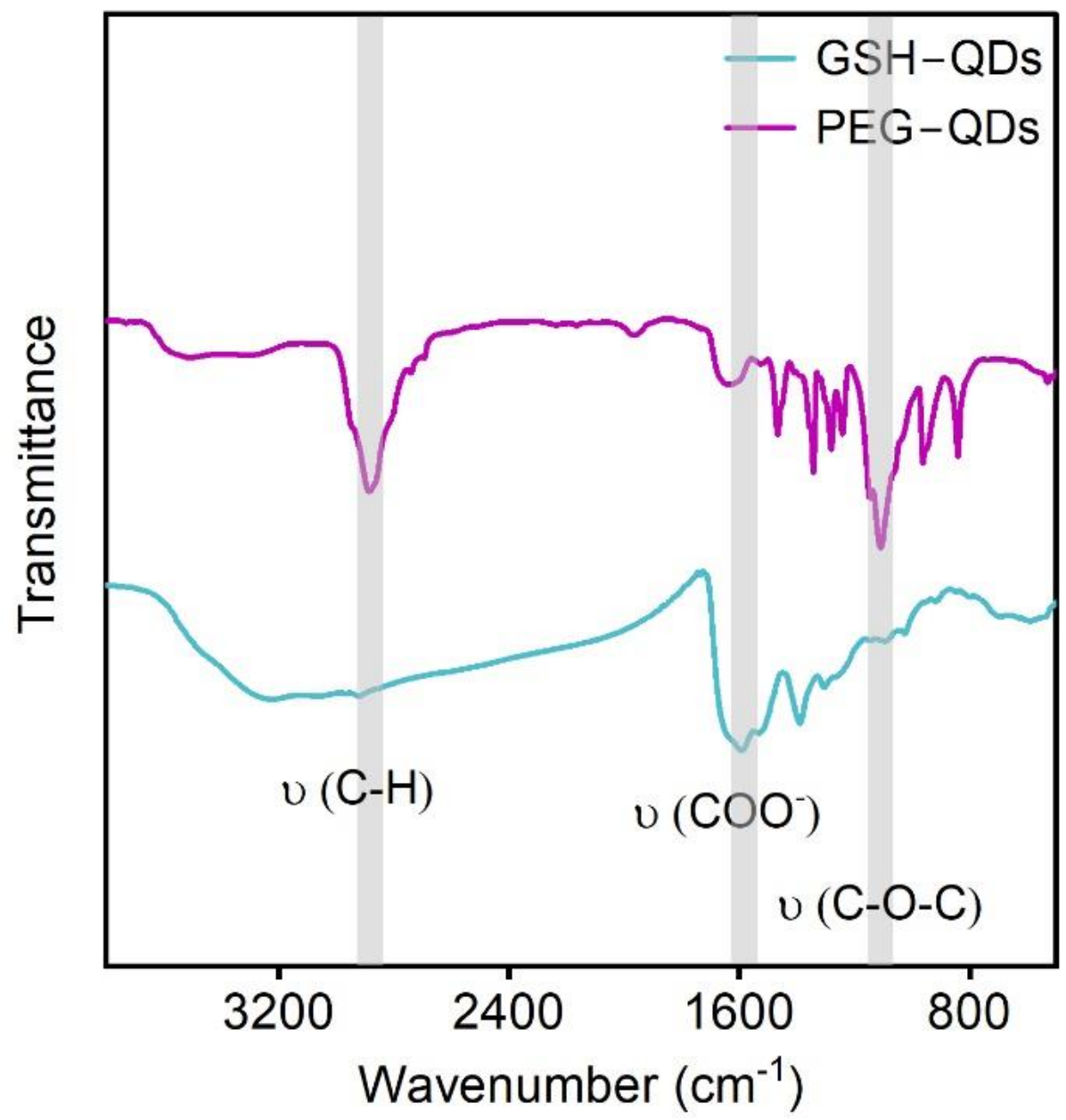

Fig. S7.

Comparison of the surface chemistry of QDs before and after ligand exchange. FTIR spectra of QDs capped with GSH and PEG, respectively.

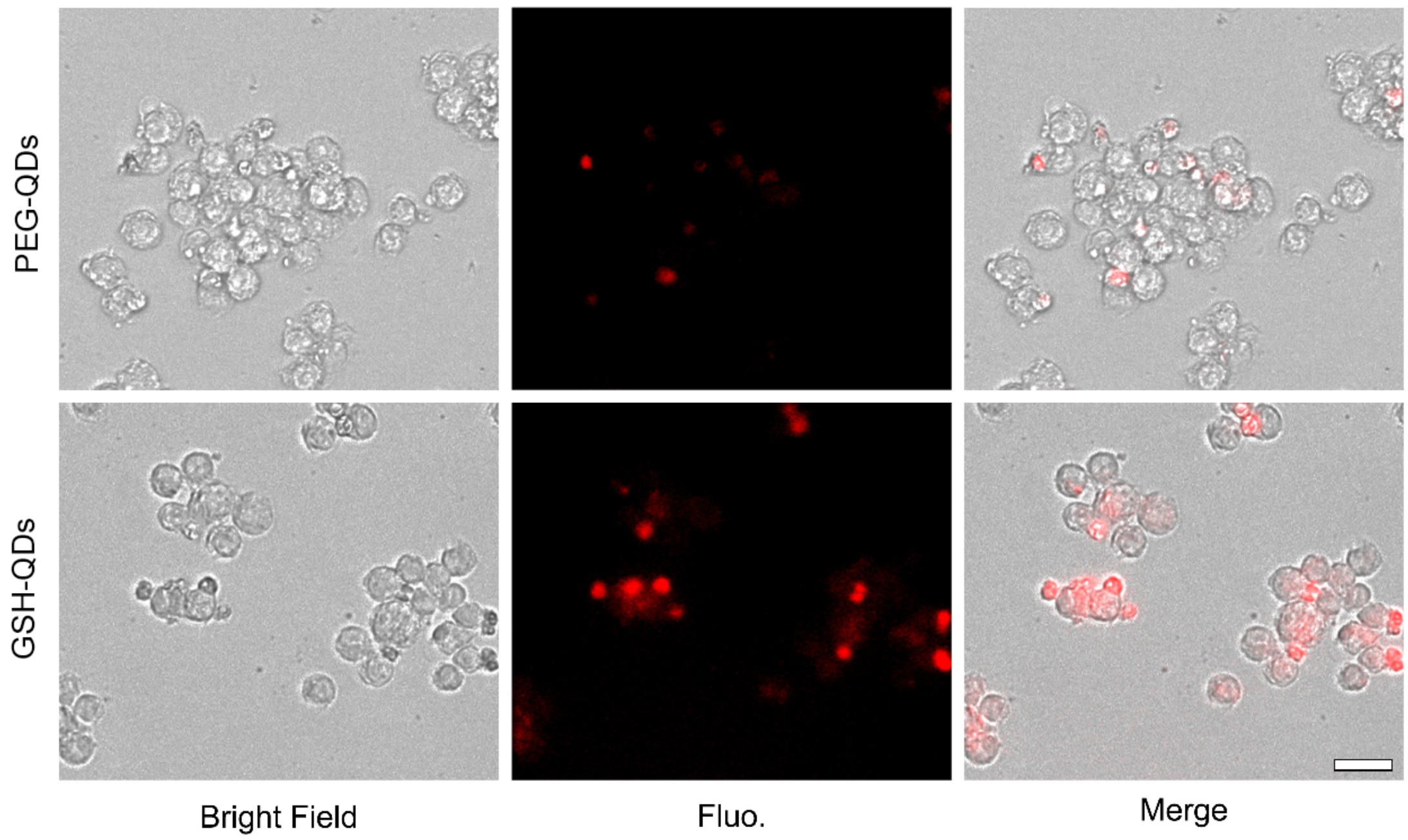

Fig. S8.

Anti-phagocytosis capability of PEG-QDs. Fluorescence images of RAW 264.7 cells following co-incubation with PEG-QDs or GSH-QDs. Scale bar: 25 μm.

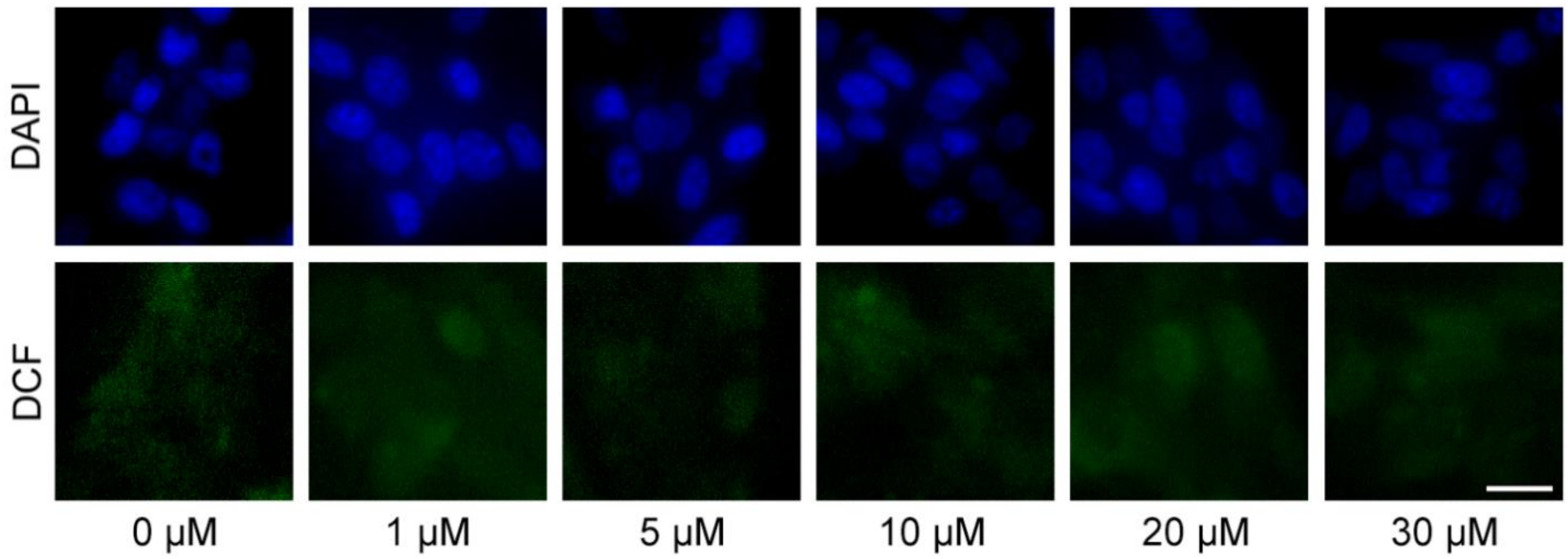

Fig. S9.

Assessment of ROS production in QD-treated cells. Fluorescence images of bEnd-3 cells treated with PEG-QDs at different concentrations, followed by DAPI staining to show the cell nuclei and DCFH-DA staining to show intracellular ROS. Scale bar: 25 µm.

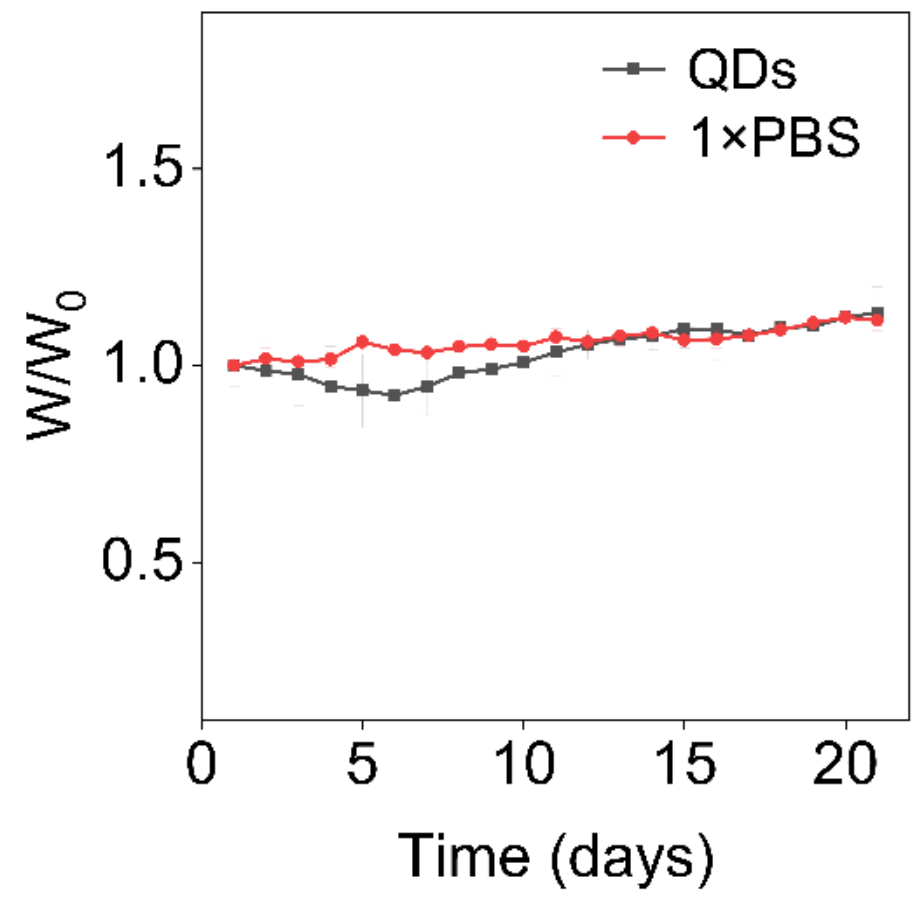

Fig. S10.

Comparison of body weight between QD-treated and control groups. Time evolutions of body weights of mice (n = 4) treated with QDs and 1× PBS, respectively.

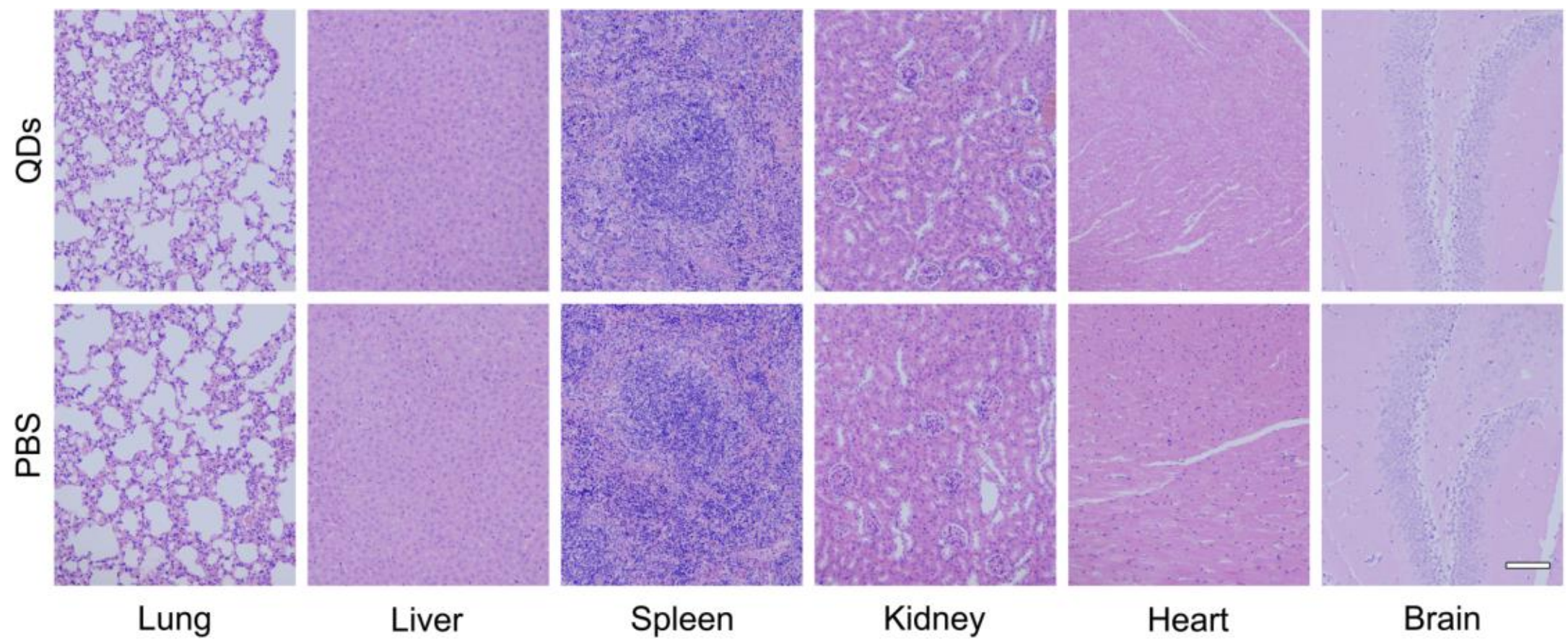

Fig. S11.

Histopathological evaluation of major organs. H&E staining of the tissue slices of major organs from QDs-treated and PBS-treated mice. Scale bar: 100 µm.

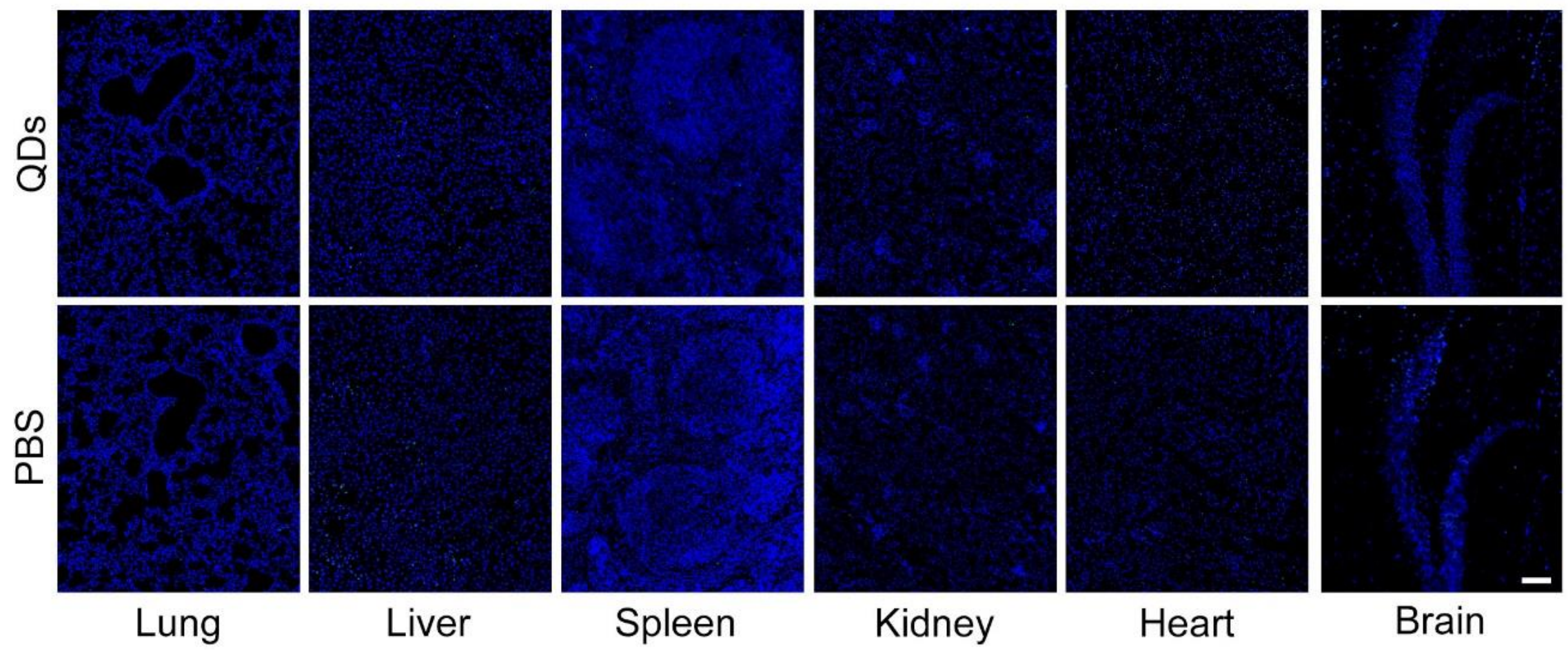

Fig. S12.

Apoptosis evaluation of major organs. TUNEL staining of the tissue slices of major organs from QDs-treated and PBS-treated mice. Scale bar: 100 μm.

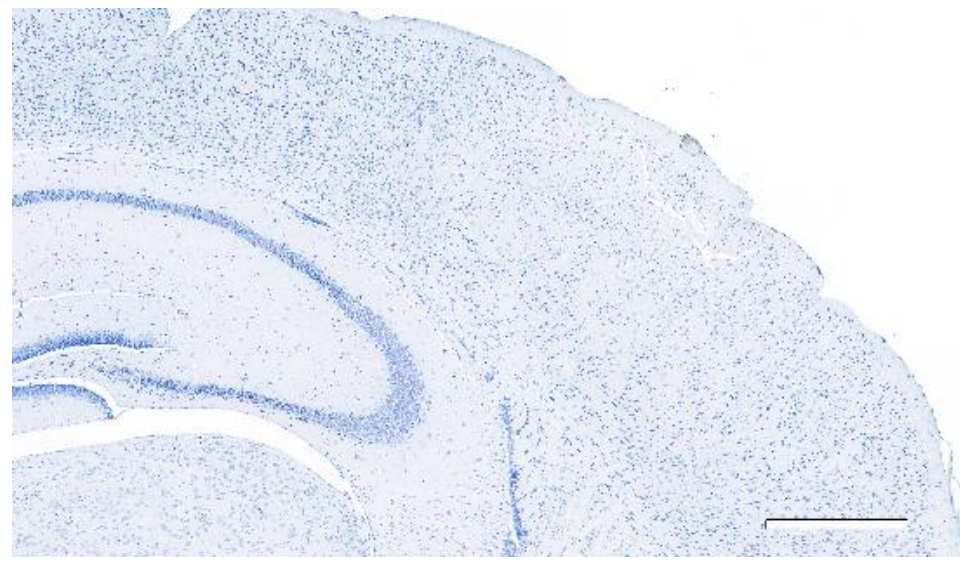

Fig. S13.

Evaluation of neuronal injury in TBI mouse brain. Nissl staining of the brain slice of TBI mouse after fluorescence imaging. Scale bar: 500 µm.

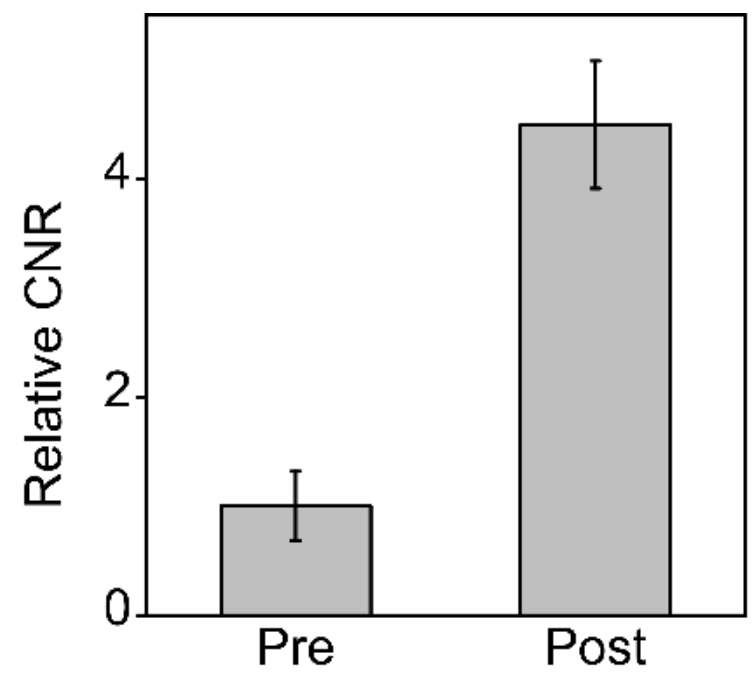

Fig. S14.

Quantitative analysis of the MR images. The CNR in the injured brain region, measured from T_1-weighted MR images acquired at 10 min after QD administration.

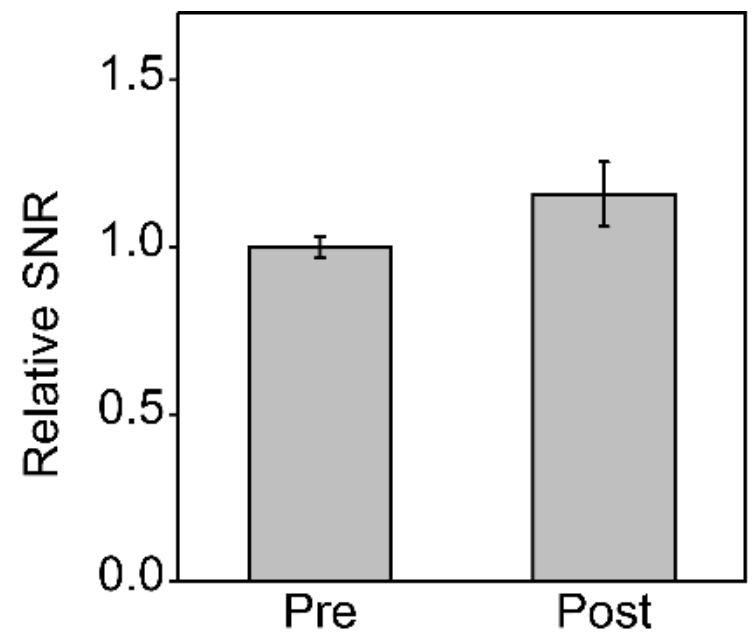

Fig. S15.

Quantitative analysis of the MR images. SNR in the injured brain region, measured from T_1-weighted MR images acquired at 10 min after QD administration.

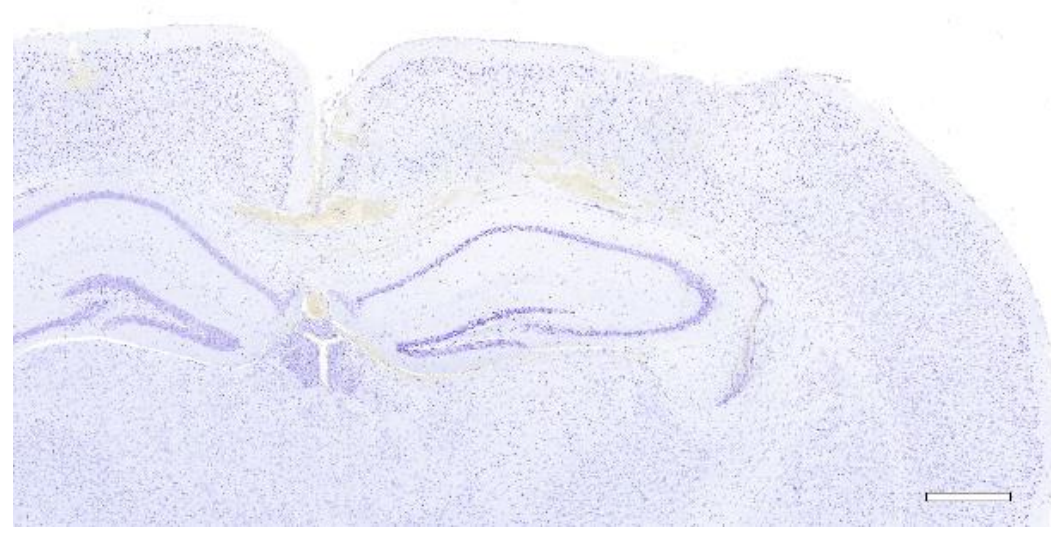

Fig. S16.

Evaluation of neuronal injury in TBI mouse brain. Nissl staining of the brain slice of TBI mouse after MRI studies. Scale bar: 500 μm.

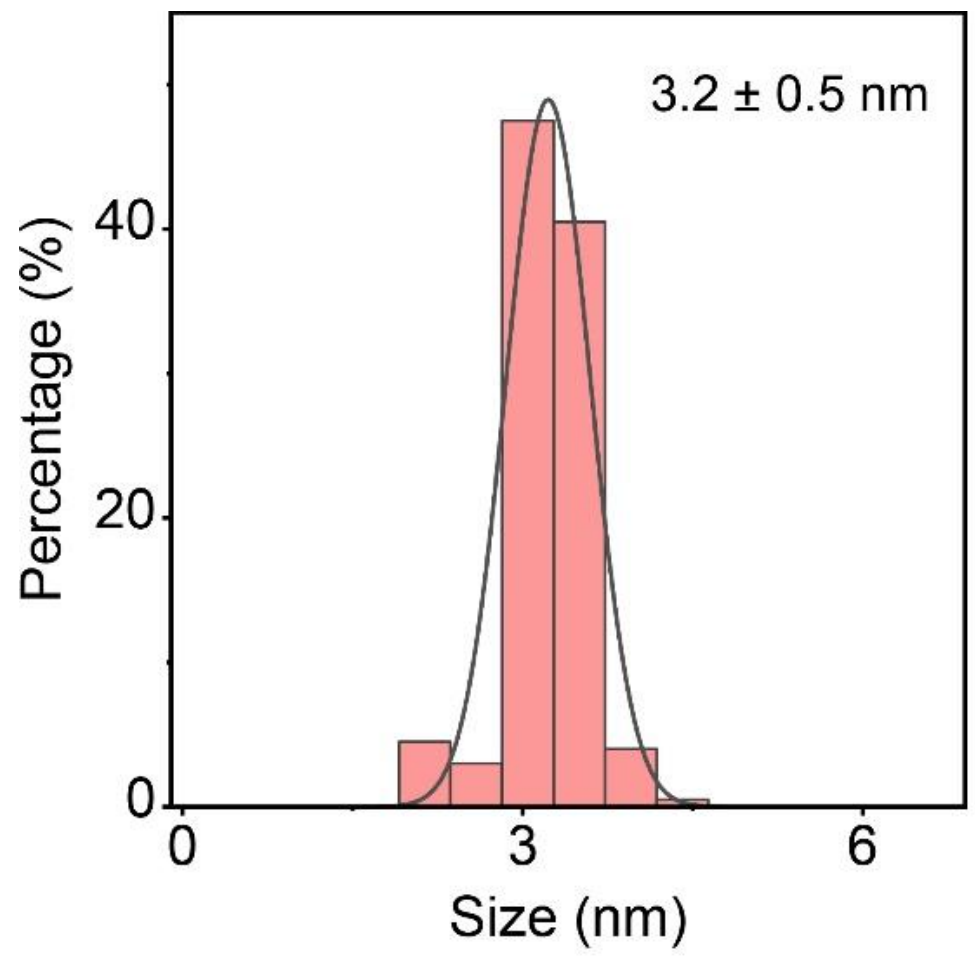

Fig. S17.
Monodispersity analysis of nanoparticles. Size histogram of PEGylated Fe_3O_4 nanoparticles.

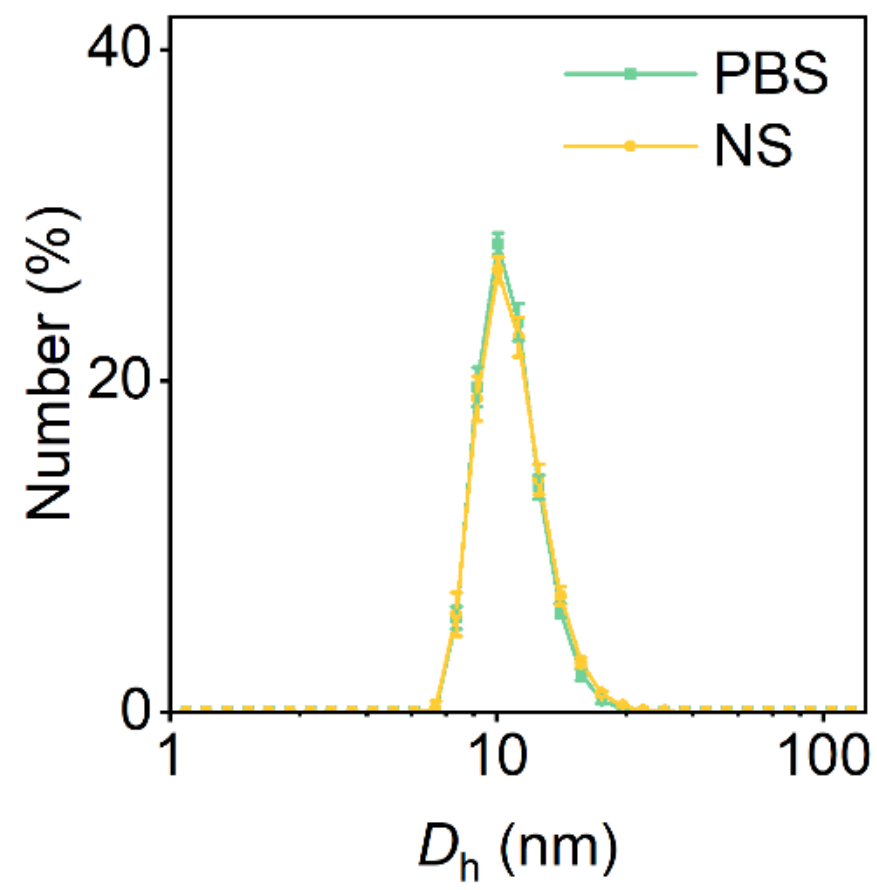

Fig. S18.

DLS analysis of colloidal nanoparticles. Hydrodynamic sizes (~10 nm) of PEGylated Fe_3O_4 nanoparticles dispersed in PBS and normal saline (NS).

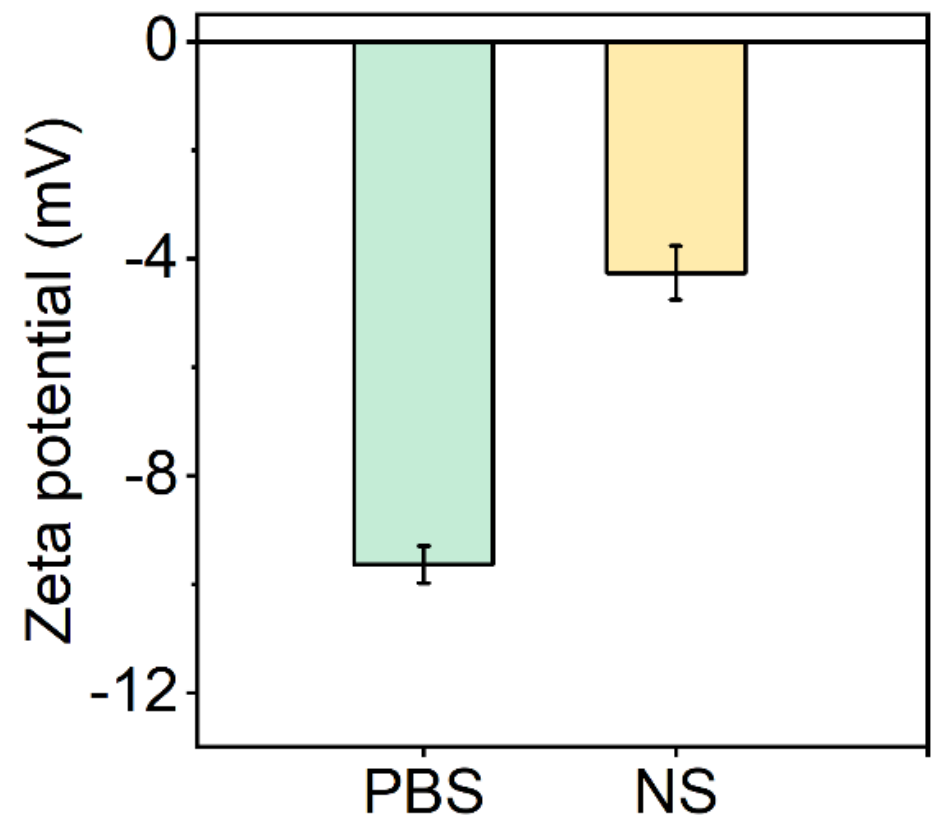

Fig. S19.

Surface charge characterizations of colloidal nanoparticles. Zeta potential of PEGylated Fe_3O_4 nanoparticles dispersed in PBS and NS.

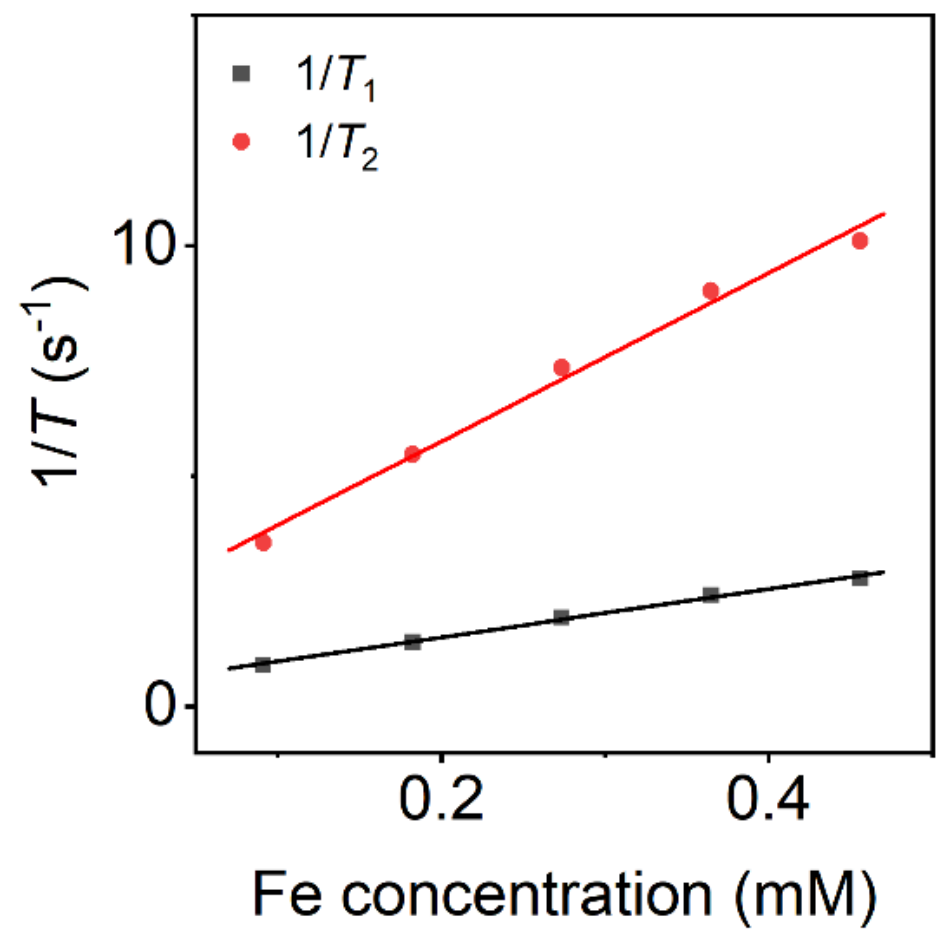

Fig. S20.

Concentration-dependent relaxivity analysis of nanoparticles. Plots of $1/T_1$ and $1/T_2$ of aqueous solutions containing PEGylated Fe_3O_4 nanoparticles of gradient concentrations versus Fe concentration overlaid with linear fitting curves for extracting the longitudinal and transverse relaxivities. The extracted r_1 and r_2 are 5.2 mM^{-1} s^{-1} and 18.2 mM^{-1} s^{-1}, respectively.

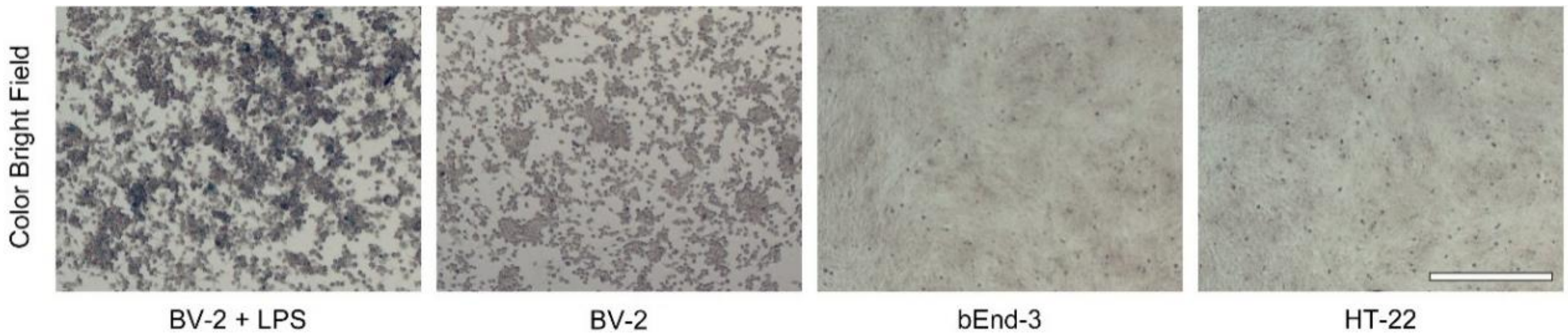

Fig. S21.

Comparison of cellular uptake of nanoparticles among different cell types. Enlarged view of Prussian blue staining of different cell types after co-incubation with PEGylated Fe_3O_4 nanoparticles. Scale bar: 500 µm.

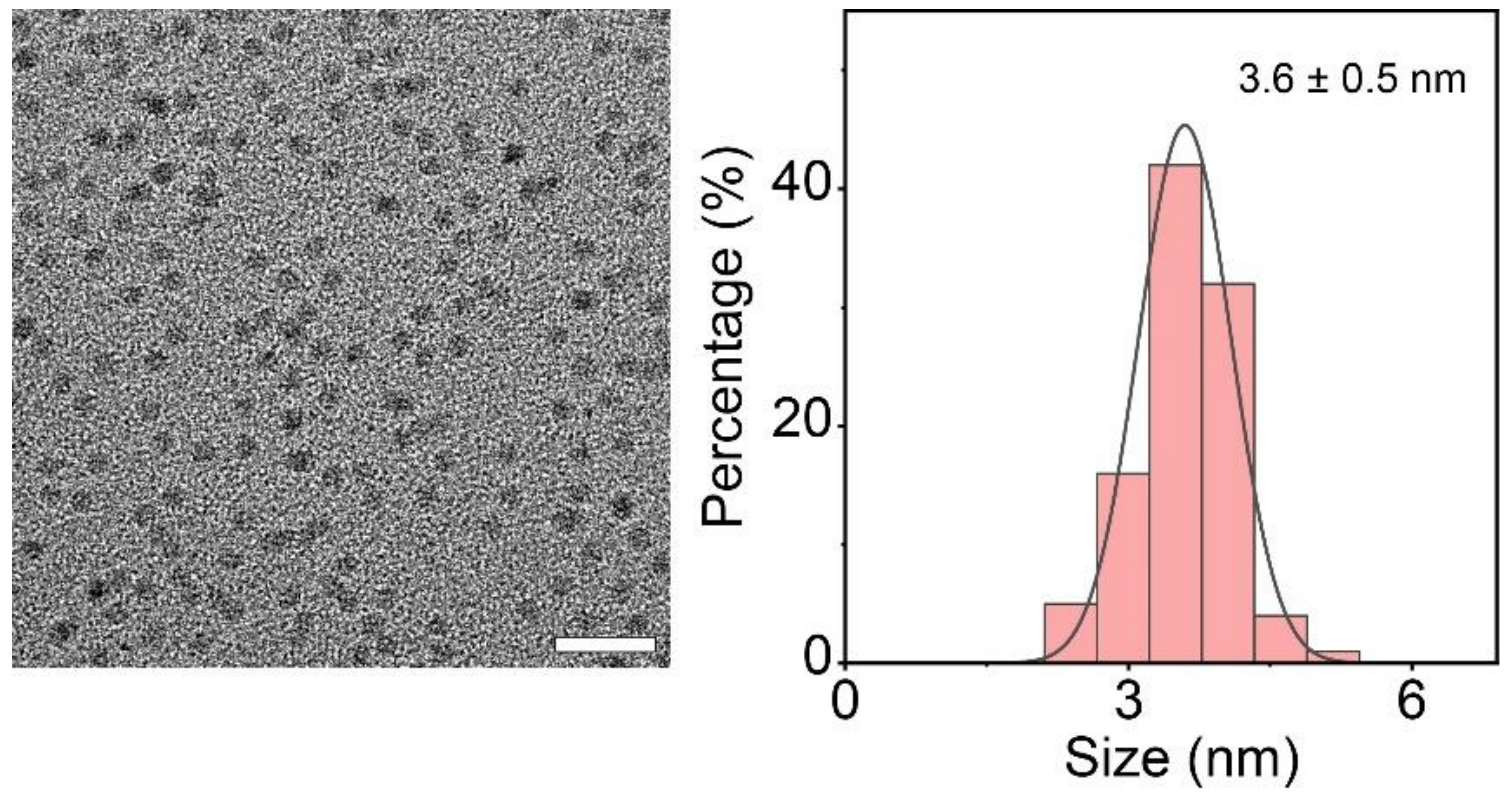

Fig. S22.

Morphology and Monodispersity analysis of nanoparticles. TEM image and size histogram of PEGylated $NaGdF_4$ nanoparticles (scale bar: 20 nm).

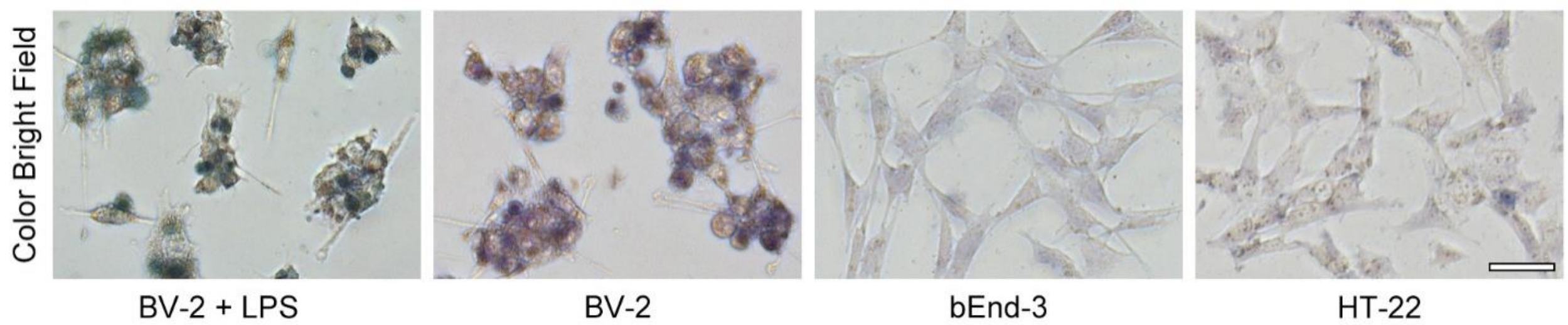

Fig. S23.

Comparison of cellular uptake of nanoparticles among different cell types.
Chlorophosphonazo III staining of different cell types after co-incubation with PEGylated $NaGdF_4$ nanoparticles (scale bar: 50 μm).

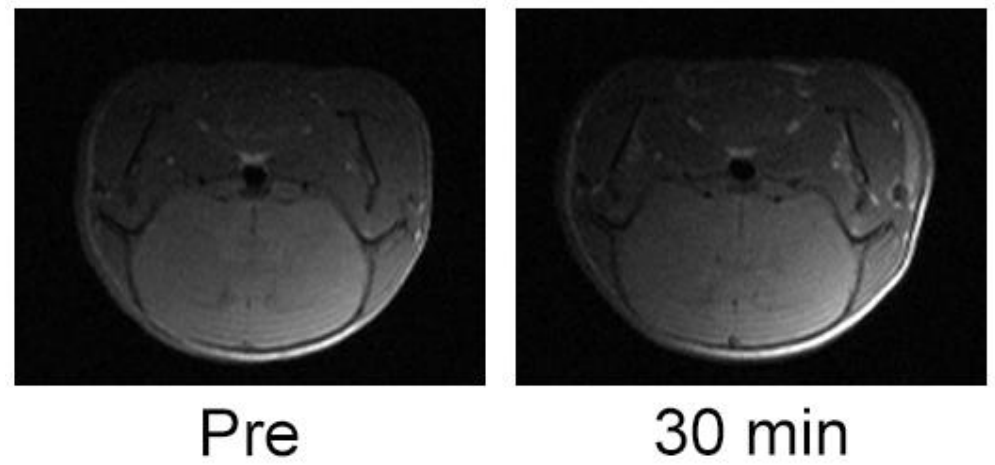

Fig. S24.

In vivo *T_1*-weighted MR images. In vivo T_1-weighted MR images of healthy mouse head recorded before and at 30 min after the intravenous injection of PEGylated Fe_3O_4 nanoparticles.

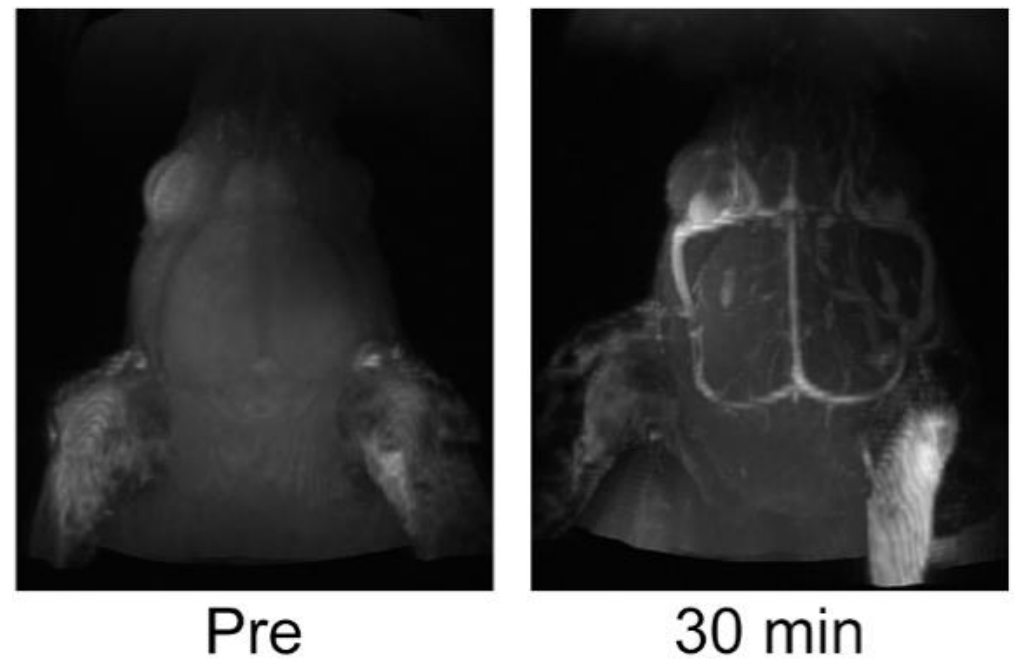

Fig. S25.

In vivo T_1-weighted MR images. DCE MR images of healthy mouse head recorded before and after the intravenous injection of PEGylated Fe_3O_4 nanoparticles.

Supplementary Tables

QD Sample	Zn:Cu:In:Mn	Mn-doping level
ZCIS@ZnS	1.00: 0.20: 3.60: 0.00	0.0%
ZCIS@ZnS:Mn	1.00: 0.32: 2.55: 0.05	1.3%
	1.00: 0.25: 2.25: 0.17	4.6%

Table S1.
The elemental composition of ZCIS@ZnS:Mn QDs with different Mn-doping levels.

Mn-doping level	λ_{em} [nm]	B_1 [%]	f_1 [%]	τ_1 [ns]	B_2 [%]	f_2 [%]	τ_2 [ns]	B_3 [%]	f_3 [%]	τ_3 [ns]	τ_{avg} [ns]	χ^2
0.0%	650	28.2	3.9	29.6	44.4	36.1	179.5	27.4	60.2	485.7	358.1	1.0
1.3%	658	41.0	6.2	21.1	38.1	30.4	118.7	20.8	63.8	456.0	328.2	1.1
4.6%	680	37.3	6.2	24.9	41.1	32.1	123.8	21.6	62.0	454.9	323.3	1.0

Table S2.

The parameters for three-exponential fitting of the PL decay curves in Fig. 1a (i.e., normalized amplitude B_i, time constant τ_i and their normalized products f_i, goodness-of-fit parameter χ^2), together with the emission peak wavelengths of QDs with different Mn doping levels.

λ_{em} [nm]	B_1 [%]	f_1 [%]	τ_1 [ns]	B_2 [%]	f_2 [%]	τ_2 [ns]	B_3 [%]	f_3 [%]	τ_3 [%]	τ_{avg} [ns]	χ^2
620	59.4	11.8	12.9	29.3	33.7	83.2	11.3	55.8	359.3	229.8	1.2
680	37.3	6.2	24.9	41.1	32.1	123.8	21.6	62.0	454.9	323.2	1.0
740	26.4	4.5	46.7	48.0	35.1	209.2	25.6	60.5	676.4	484.8	1.1

Table S3.

The parameters for three-exponential fitting of the PL decay curves in Fig. 1a (i.e., normalized amplitude B_i, time constant τ_i and their normalized products f_i, goodness-of-fit parameter χ^2), for different emission wavelengths of QDs with the Mn doping level of 4.6%.

Supplementary Movies

Movie S1: 3D panoptic immunofluorescence imaging of the entire brain.

Movie S2: 3D dynamic contrast enhanced (DCE) MRI.
